# Enhanced transcriptome maps from multiple mouse tissues reveal evolutionary constraint in gene expression

**DOI:** 10.1038/ncomms6903

**Published:** 2015-01-13

**Authors:** Dmitri D. Pervouchine, Sarah Djebali, Alessandra Breschi, Carrie A. Davis, Pablo Prieto Barja, Alex Dobin, Andrea Tanzer, Julien Lagarde, Chris Zaleski, Lei-Hoon See, Meagan Fastuca, Jorg Drenkow, Huaien Wang, Giovanni Bussotti, Baikang Pei, Suganthi Balasubramanian, Jean Monlong, Arif Harmanci, Mark Gerstein, Michael A. Beer, Cedric Notredame, Roderic Guigó, Thomas R. Gingeras

**Affiliations:** 1 Bioinformatics and Genomics Programme, Centre for Genomic Regulation (CRG) and UPF, Doctor Aiguader, 88, Barcelona 08003, Spain; 2 Faculty of Bioengineering and Bioinformatics, Moscow State University, Leninskie Gory 1-73, 119992 Moscow, Russia; 3 Functional Genomics Group, Cold Spring Harbor Laboratory, 1 Bungtown Road, Cold Spring Harbor, New York 11724, USA; 4 Faculty of Chemistry, Institute for Theoretical Chemistry, University of Vienna, Waehringerstrasse 17, 1090 Vienna, Austria; 5 Program in Computational Biology and Bioinformatics, Yale University, 266 Whitney Avenue, New Haven, Connecticut 06520, USA; 6 Department of Molecular Biophysics and Biochemistry, Yale University, 266 Whitney Avenue, New Haven, Connecticut 06520, USA; 7 Department of Human Genetics, McGill University, Montreal, Canada H3A 1B; 8 Department of Computer Science, Yale University, 51 Prospect Street, New Haven, Connecticut 06511, USA; 9 Department of Biomedical Engineering and McKusick-Nathans Institute of Genetic Medicine, Johns Hopkins University, Broadway Research Building 573, Baltimore, Maryland 21205, USA

## Abstract

Mice have been a long-standing model for human biology and disease. Here we characterize, by RNA sequencing, the transcriptional profiles of a large and heterogeneous collection of mouse tissues, augmenting the mouse transcriptome with thousands of novel transcript candidates. Comparison with transcriptome profiles in human cell lines reveals substantial conservation of transcriptional programmes, and uncovers a distinct class of genes with levels of expression that have been constrained early in vertebrate evolution. This core set of genes captures a substantial fraction of the transcriptional output of mammalian cells, and participates in basic functional and structural housekeeping processes common to all cell types. Perturbation of these constrained genes is associated with significant phenotypes including embryonic lethality and cancer. Evolutionary constraint in gene expression levels is not reflected in the conservation of the genomic sequences, but is associated with conserved epigenetic marking, as well as with characteristic post-transcriptional regulatory programme, in which sub-cellular localization and alternative splicing play comparatively large roles.

## Abstract (web summary)

The analysis of mammalian transcriptomes could provide new insights into human biology. Here the authors carry out RNA sequencing in a large collection of mouse tissues and compare these data to human transcriptome profiles, identifying a set of constrained genes that carry out basic cellular functions with remarkably constant expression levels across tissues and species.

## Graphical abstract

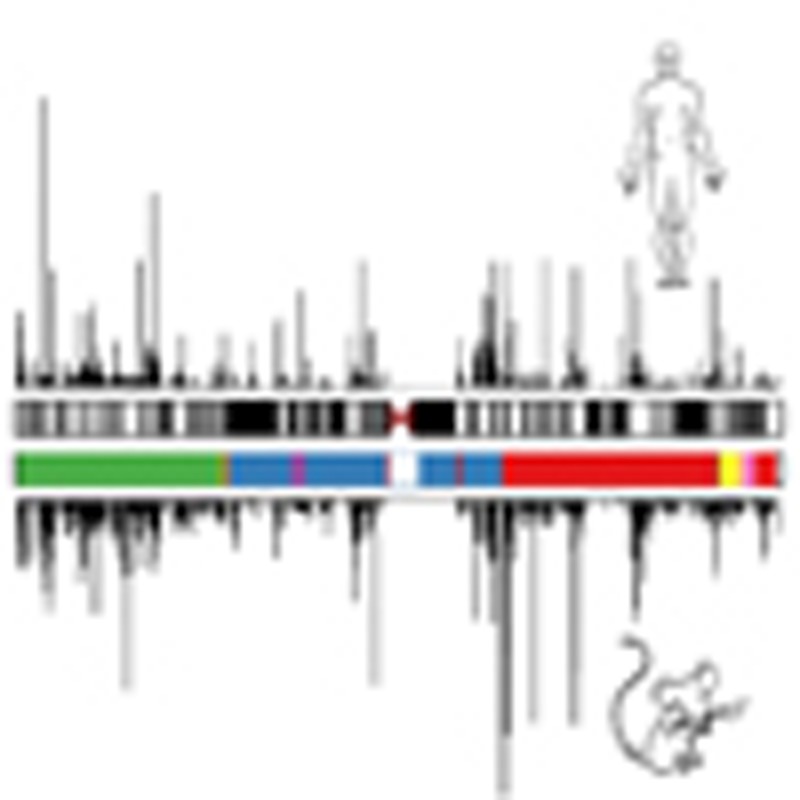

Approximately 90 million years of evolution separate the mouse and the human genomes. During this period, selected and neutral genetic changes have accumulated, resulting in 60% nucleotide divergence^1^. Structural and coding organization, however, have been substantially maintained with approximately 90% of the mouse and human genomes partitioning into regions of conserved synteny, and more than 15,000 protein-coding orthologues (about 80% of all protein-coding genes) shared between these two genomes^2^,^3^. Substantial information on the functional elements encoded in the human genome has been accumulated over the years. However, despite considerable effort^4^,^5^, the mouse genome remains, in comparison, poorly annotated.

Here we characterize the transcriptional profiles from a diverse and heterogeneous collection of fetal and adult mouse tissues by RNA sequencing (RNA-seq). Using this data in conjunction with other data recently published^6^, we extend the mouse gene and transcript candidate set, and enhanced the current set of orthologous genes between these genomes to include long non-coding RNAs (lncRNAs) and pseudogenes. We also compare the mouse expression profiles with expression profiles in human cell lines obtained in the framework of the ENCODE project, using identical sequencing and analysis protocols^7^,^8^. Although the compared profiles do not correspond to matched biological conditions, preventing the investigation of the evolutionary conservation of cell type versus species-specific transcriptional patterns, they allow for an investigation of the conservation of transcriptional features that are independent of the cell types specifically monitored. In particular, we have identified a well-defined subset of genes, the expression of which remains relatively constant across the disparate mouse tissues and human cell lines investigated here. Comparison with transcriptional profiles in multiple tissues of other vertebrate species^9^,^10^ reveals that the constraint in expression has likely been established early in vertebrate evolution. Genes with constrained expression capture a relatively large and constant proportion of the RNA output of differentiated cells but not of undifferentiated cells, and is the main driver of the notable conservation of transcriptional profiles reported between human and mouse^2^,^11^,^12^ and other mammals^13^. Our analysis further shows that these genes are under specific conserved transcriptional and post-transcriptional regulatory programmes.

## Results

### Expanded mouse transcriptional annotations

A total of 30 mouse embryonic and adult tissue samples and 18 human cell lines (generated as part of the human ENCODE project^7^) were used as sources for the isolation of polyadenylated (polyA+) long (>200 nucleotides (nt)) RNAs (Supplementary Table 1), which were sequenced in two biological replicates to an average (AVG) depth of 450 million reads per sample. Sequence reads were mapped and post-processed to quantify annotated elements in GENCODE^14^ (human v10, hg19) and ENSEMBL^15^ (mouse ens65, mm9), and to produce *de novo* transcriptional elements as previously described^8^. Reproducibility between replicates was assessed using a non-parametric version of the Irreproducible Discovery Rate (IDR) statistical test^8^ (Supplementary Methods and Supplementary Tables 2,3A, and 3B).

**Figure 1:**
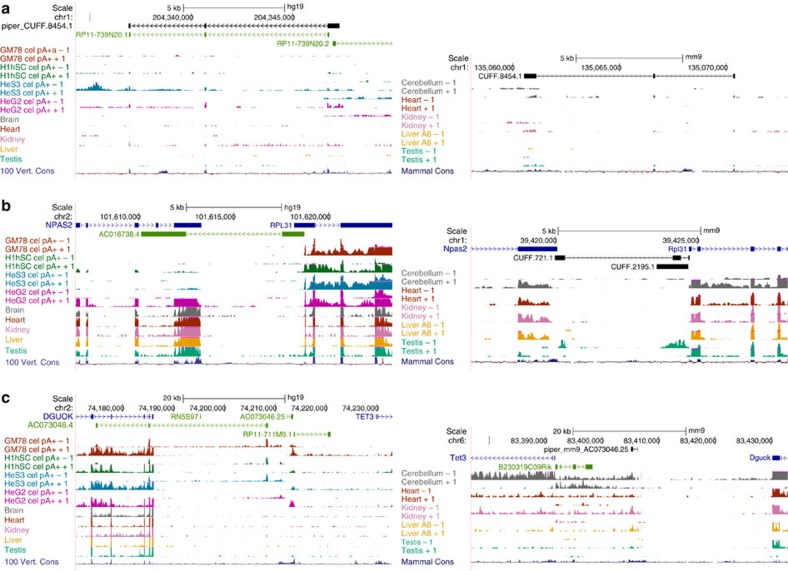
Examples of extensions to the mouse annotation (left: human; right: mouse). (**a**) The novel intergenic transcript model CUFF.8454.1 has been inferred from mouse RNA-seq data in a region of the mouse genome without gene annotations. Mapping of this transcript model to the human genome reveals that it is the homologue of the lncRNA RP11-739N20.1 annotated in the human genome. (**b**) Two mouse transcript models, CUFF.721.1 and CUFF.2195.1, have predicted antisense to two neighbouring protein-coding loci (Npas2 and RPL31). RNA-seq shows that their expression is restricted to testis. CUFF.721.1 is the likely homologue of the annotated human ACO16738.4, which is antisense and overlaps the human orthologue of Npas2 and RPL31. In human, ACO16738.4 appears to be also expressed specifically in testis, although this is not conclusive, as we lack stranded RNA-seq data. (**c**) The mapping of the human AC0703046.25 lncRNA to the mouse genome identified a potential mouse transcript in the syntenic region between the DGUOK and the TET3 genes (piper_mm9_AC073046.25). This transcript was not included in our predicted models, but it has strong support by RNA-seq data, specifically in cerebellum, kidney and liver. Tissue RNA-seq data in human are from Ilumina Body Map HBM. Plots are UCSC browser screenshots where novel mouse models are indicated in black, human gencode annotation in blue, green and red, and mouse CSHL and human HBM RNA-seq signal in different colours at the bottom. Annotated genes are represented by the longest transcript.

Reflecting the less developed state of the annotation of the mouse genome, GENCODE (v10) includes 164,174 long human transcripts, compared with 90,100 long mouse transcripts included in ENSEMBL (v65). By combining transcript predictions obtained using Cufflinks^16^ in our sequenced RNA samples with cap analysis of gene expression (CAGE) tag clusters recently produced by the FANTOM project^6^, we have identified about 150,000 novel transcripts in human^8^, and 200,000 in mouse (Supplementary Table 3B), leading to similar numbers of transcripts in the two species, as illustrated by a few examples in Fig. 1 (Supplementary Methods and Supplementary Table 3C). In addition, the mapping of the novel mouse transcripts back to the human genome led to the discovery of 38 novel human genes not included in the models derived from human RNA-seq data, but supported by CAGE clusters. This underlines the importance of comparative approaches in completing genome annotations. By directly using the split RNA-seq reads at a stringent entropy threshold (Supplementary Methods), we identified a set of about 400,000 highly confident splice junctions in the mouse genome, of which about half are novel. In contrast to annotated junctions, novel junctions are highly tissue-specific (Supplementary Fig. 1 and Supplementary Table 4A). By comparing to splice junctions in human, and using one-to-one whole-genome maps^17^, we have assembled a set of 204,887 orthologous splice junctions (Supplementary Table 4B). Moreover, we combined genome annotations and RNA-seq evidence and identified 3,641 mouse genes with antisense transcription (Supplementary Methods)—a proportion of which, larger than expected by chance, are orthologous to human genes with antisense transcription (χ^2^-test, *P*-value<10^−16^; Supplementary Table 5), indicating conservation in the occurrence of antisense transcription at human–mouse orthologous loci^18^ (Fig. 1b). Examples in Supplementary Fig. 2 and Supplementary Table 6 show potential novel cases of a regulatory mechanism recently described involving antisense lncRNAs that contain a short interspersed nuclear element (SINE)^19^.

Using one-to-one whole-genome maps and sequence homology, we identified a set of 851 human–mouse orthologous lncRNA genes (Fig. 1, Supplementary Methods and Data). Of these, only 189 overlap with the set of 2,736 one-to-one human–mouse lncRNAs recently described^20^, reflecting the yet incomplete characterization of mammalian lncRNAs, and of lncRNA orthology. Using localization data in human cell lines^8^, we found these genes to segregate in two clearly distinct nuclear versus cytosolic populations (Supplementary Fig. 3A). Expression levels of orthologous lncRNAs correlate weakly with phylogenetic depth (that is, the number of mammalian species in which a given lncRNA can be detected, Supplementary Fig. 3B). Although lncRNAs show distinct tissue- and species-specific expression patterns, we identified 12 lncRNAs expressed in at least 50% of the samples in each species. This small set of conserved broadly expressed genes may play important functions in mammalian cells (see Fig. 1c for an example). We found these genes to be highly enriched among nuclear lncRNAs (Supplementary Fig. 3A). In addition, we identified a set of 129 orthologous pseudogenes, of which 32% are expressed in one species and 4% in both (Supplementary Methods).

### Genome-wide conservation of gene expression and splicing

**Figure 2:**
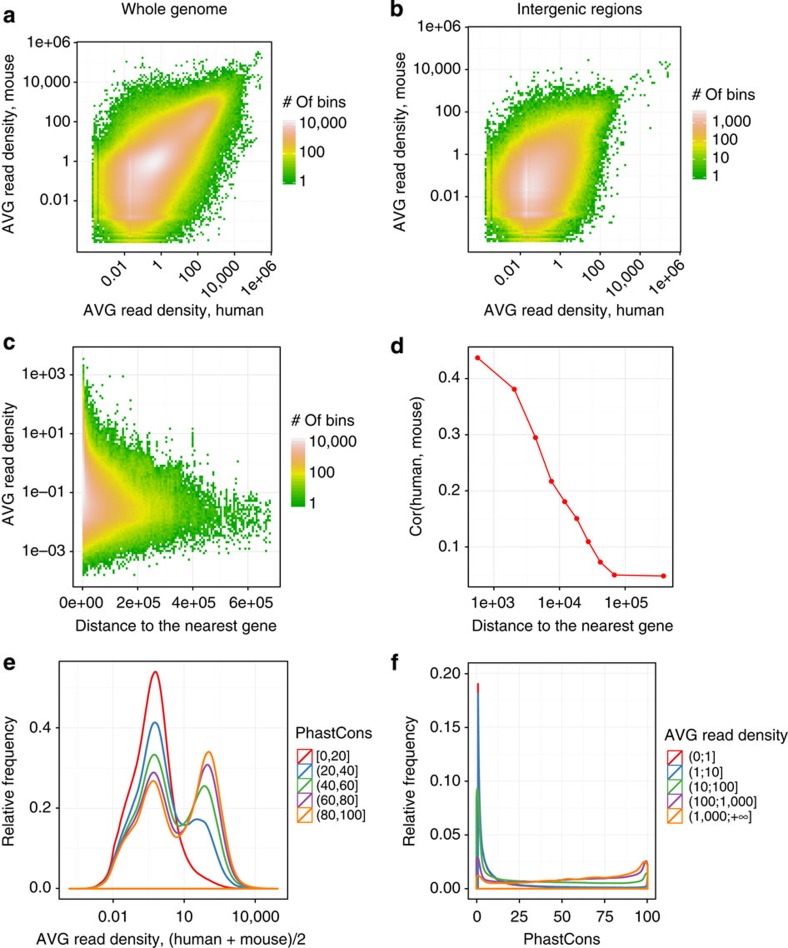
Genome-wide conservation of expression profiles. (**a**) The joint distribution of log_10_ average (AVG) read density in orthologous 100-nt bins in human (*x*-axis) and in mouse (*y*-axis); cc=0.67. (**b**) The distribution in **a** limited to intergenic regions; cc=0.37. (**c**) The distribution of log_10_ average read density in intergenic regions (average between human and mouse) as a function of the distance from the nearest gene. (**d**) Correlation of log_10_ average read density in human and mouse as a function of distance from the nearest gene. (**e**) The distribution of log_10_ average read density in 100-nt bins as a function of phastCons score (conservation score across 45 vertebrate species). (**f**) The distribution of phastCons score of a 100-nt bin as a function of the average read density.

**Figure 3:**
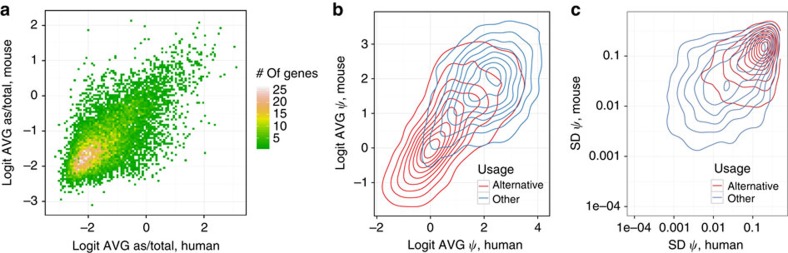
Genome-wide conservation of antisense expression and splicing profiles. (**a**) The joint distribution of the average antisense-to-total expression ratio (the number of reads mapped to the opposite strand as a fraction of the number of reads mapped to both strands) in pairs of orthologous protein-coding genes; cc=0.68. (**b**,**c**) Contour plots of the joint probability distribution of the average usage (*ψ*, per cent-spliced-in) of splice junctions (SJ; **b**), and of the standard deviation of SJ usage (**c**) in orthologous SJ pairs. Logistic transformation (*logit*) was used in **a** and **b**. SJ with constant complete inclusion or exclusion are not shown. ‘Alternative’ denotes SJ that are annotated alternative in both species.

There is overall, substantial genome-wide conservation of expression levels between human and mouse irrespective of the cell or tissue type of the sample. We computed genome-wide expression profiles, measured as AVG read density, for all orthologous 100-nt bins spaced equally along the human and mouse genomes (Supplementary Fig. 4A,B, and Supplementary Methods). We found substantial correlation in AVG read density at orthologous bins (Pearson correlation coefficient, cc=0.67, Fig. 2a). This correlation is significant not only for exonic regions^2^ (Supplementary Fig. 4C), but also for alignable intronic (Supplementary Fig. 4D) and intergenic regions (Fig. 2b). However, most of this intergenic transcription is proximal to annotated genes (41% less than 10 kb from the closest annotated gene termini, Fig. 2c). This is partially the consequence of the decreasing number of intergenic bins with distance to the closest gene (Supplementary Fig. 5). In any case, the murine-human expression correlation decays with distance to the closest annotated gene (Fig. 2d). Permissive transcription close to protein-coding genes could be the origin of many lncRNAs. However, when computing the distance between annotated lncRNAs and the closest neighbour annotated gene, we found this distance to be larger than for protein-coding genes (on AVG, approximately 66 and 35 kb, respectively). Expression levels correlate with phylogenetic conservation, as measured by phastCons scores^21^,^22^ (Fig. 2e). However, a fraction of orthologous bins having low sequence conservation are still densely transcribed (5% of the least conserved bins have read density greater than 10) and the bins that correspond to higher expression include a wide range of sequence conservation values (Fig. 2f). Highly expressed intergenic bins are slightly enriched for genome-wide association study (GWAS) hits (Fisher test, *P*-value≈0.055), and strongly enriched for *cis*-expression quantitative trait locus (QTLs) (eQTLs; *P*-value<2.2e-16, Supplementary Methods), the latter suggesting an important role for enhancer transcription in the regulation of gene expression. There is also substantial conservation of antisense transcription^18^. For each sense/antisense orthologous gene pair, we computed the ratio of antisense-to-total gene expression averaged over all conditions, and found strong correlation of AVG antisense-to-total gene expression ratio in orthologous genes (cc=0.68) as well as of its variation among samples (cc=0.52; Fig. 3a and Supplementary Fig. 6). Antisense transcription has been suggested as an important regulatory mechanism^23^, and our results indicate that it may have been conserved over large evolutionary distances.

Conservation of the exonic structure of human–mouse orthologous genes, as well as of the splice site sequences, has also been reported^24^. RNA-seq further allows the investigation of the patterns of usage of exons and splice junctions. We computed the AVG inclusion, measured as percent spliced-in (*ψ*), and found strong correlation of inclusion at orthologous splice junctions (cc=0.59, Fig. 3b). The correlation is mostly driven by relatively low-AVG inclusion levels of junctions annotated as alternative in both human and mouse. These junctions constitute a large part of the orthologous set (45%) and are used more variably in both species (Fig. 3c). In addition, we computed AVG splicing processivity, measured using the completeness of splicing index (*θ* (refs 25, 26)), and found also significant correlation, albeit lower (cc=0.35, Supplementary Fig. 7A,B). This is expected, as the inclusion level of an exon in the final RNA product is likely to be physiologically more relevant than the efficiency with which the exon is included.

### Evolutionary conserved constraint in gene expression

**Figure 4:**
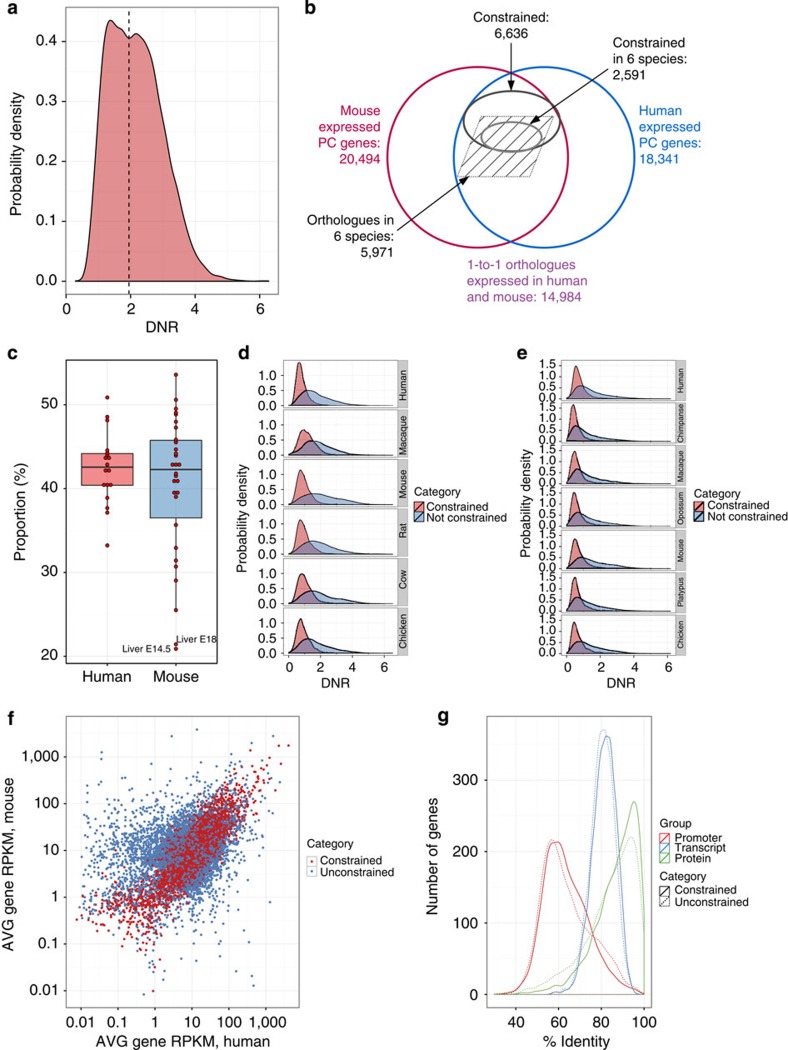
Genes with constrained expression. (**a**) The distribution of the dynamic range (DNR, log_10_ of the ratio of the largest and the lowest non-zero observation) of gene expression level in orthologous genes across human and mouse samples. (**b**) Venn diagram of the relationship between orthologous and constrained genes. (**c**) Proportion of nucleotides in expressed genes, as assessed by PolyA+ RNA-seq, that originates from constrained genes in human cell lines and mouse tissues. The labelled outliers correspond to mouse embryonic samples. (**d**,**e**) The distribution of DNR in human/mouse constrained and unconstrained genes in Merkin *et al.*^10^ (**d**) and Barbosa-Morais *et al.*^9^ (**e**). (**f**) The joint distribution of log_10_ average gene reads per kilobase per million mapped reads (RPKM) in pairs of orthologous protein-coding genes; constrained genes are shown in red. (**g**) The distribution of promoter, transcript and protein pairwise sequence identity between human and mouse in constrained and unconstrained genes.

As previously reported^8^,^27^, we found that within a given cell population the levels of gene expression may vary up to six orders of magnitude (Supplementary Fig. 8). In contrast, however, the expression of a given gene across cell types varies relatively little. Using a two-factor analysis of variance (ANOVA), with gene and cell type as two factors, we found that the variance across genes accounts for 76% and 71% of the total variance of gene expression across human and mouse cell types, respectively, whereas the fraction of the variance that can be directly attributed to cell type is less than 1% (Supplementary Methods). Indeed, we found a large fraction of genes, the expression of which varies relatively little across tissues and species. In Fig. 4a, we computed the distribution of the dynamic range of gene expression (DNR) in orthologous genes across the entire set of human and mouse samples. For each gene expressed in at least two samples both in human and mouse, we computed the log_10_ ratio between the highest and lowest measured expression. The distribution is bimodal, uncovering two broad gene classes. This is not an effect of comparing mouse tissues with human cell lines, as the same pattern is obtained when using RNA-seq obtained in human tissues by the Illumina Body Map project (Supplementary Fig. 9). We decompose the distribution assuming two underlying Gaussians, and we took DNR=2 at the approximate intersection point (Supplementary Fig. 10 and Supplementary Methods). In this way, we obtained a set of 6,636 genes (~40% of all 15,736 orthologous genes, Fig. 4b), the expression of which remains relatively constant (that is, within two orders of magnitude) across species and cell types. Genes with constrained expression show wider expression breadth (Supplementary Fig. 11A) and less tissue specificity than the rest of the orthologues (less than 10% of tissue-specific orthologues are included in this set, Supplementary Methods). However, they can be eventually detected as differentially expressed at a rate similar to the rest of the orthologues (82% versus 89%). They also show higher expression levels (Supplementary Fig. 11B). Therefore, although they represent only about 17% of all annotated genes, they capture a high proportion of the polyA+ transcription in the cell (39% on AVG in human and 41% in mouse), a proportion that remains remarkably constant across all tested human and mouse cell types (Fig. 4c). Mouse embryonic samples are an exception, with constrained genes generating only about 20% of the cell’s transcriptional output. We also found a negative association between minimal expression and DNR (Supplementary Fig. 12). Thus, genes with constrained expression tend to have also higher minimal expression, suggesting that these genes in their default state may already be primed for transcription. To eliminate gene expression as a potential confounding factor, most downstream analyses have been carried out in a subset of 5,519 genes with constrained expression, and in a subset of identical size from the rest of the orthologous genes for which we could compute DNR, with matched expression in human and mouse (the unconstrained set, Supplementary Fig. 11C and Supplementary Methods).

Using transcriptome data recently published in multiple tissues across a number of vertebrate species^9^,^10^, we found that the expression of these genes is actually constrained beyond human and mouse, and seems to have been established early in vertebrate evolution. About 94–98% of genes with constrained expression in human and mouse have DNR across tissues less than two in other vertebrate species (Fig. 4d,e). We further identified a collection of 2,591 genes whose expression levels show relatively little variation across different cell types and species within vertebrates (out of the 5,971 orthologs across these species that are expressed in both mouse and human^10^) (Fig. 4b). Gene Ontology analysis reveals that genes with constrained expression participate in basic functional and architectural housekeeping processes common to all cell types (Supplementary Data archive 5). These genes are the main drivers of the substantial conservation of expression reported in orthologous human and mouse genes^28^,^29^,^30^. Indeed, the correlation of AVG gene expression between human and mouse measured in the set of constrained genes is 0.82, compared with only 0.32 for the unconstrained gene set (Fig. 4f).

**Figure 5:**
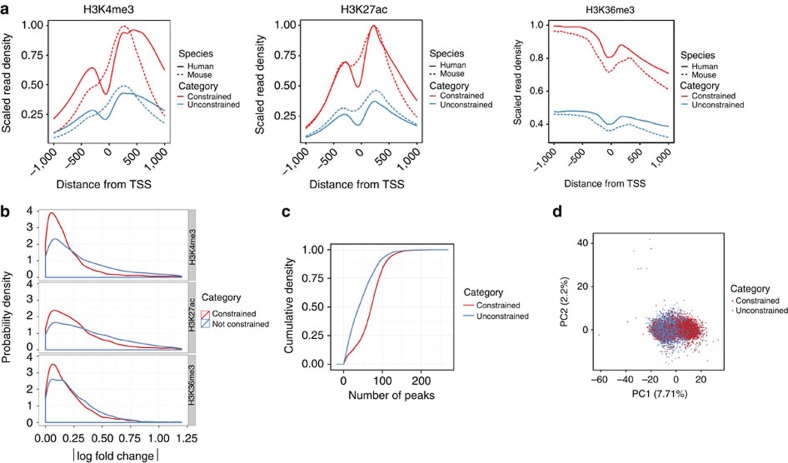
Conservation of epigenetic marks in constrained genes. (**a**) Normalized histone modification profiles 1 kb around transcription start site (TSS) for H3K4me3 and H3K27ac, and around the TTS for H3K36me3 in constrained and unconstrained genes. (**b**) The difference in normalized average histone modification signals 1 kb around TSS (|Δlog Signal|) in constrained and unconstrained genes. (**c**) The cumulative distribution of the number of TF ChIP-seq peaks in promoter regions of human constrained and unconstrained genes. (**d**) Principal component analysis of ChIP-seq measured binding strength of TFs in constrained and unconstrained genes.

Constraint of gene expression is not reflected in sequence conservation of either the gene body or the proximal promoter regions^31^ (Wilcoxon test, *P*-value≈0.11 for the difference in phastCons scores; Fig. 4g). In contrast, we found that constrained genes exhibit characteristic patterns of histone modifications, quite divergent from that of unconstrained genes. Using the data collected in these and the human ENCODE studies^3^,^32^ (Supplementary Table 7A,B and Supplementary Methods), we computed genic profiles of normalized histone modification signals, averaging them over all studied cell types (Fig. 5a). We found much stronger signal for active histone modifications (H3K4me3, H3K27ac and H3K36me3) in constrained compared with unconstrained genes. As the set of genes in which we performed the analysis are controlled for differences in gene expression, this is not the result of constrained genes exhibiting higher expression levels. Furthermore, we controlled by gene expression within each sample separately, and found the same effect (Supplementary Fig. 13). Although the association between gene expression and chromatin structure is well known, and models have been developed able to predict gene expression from levels of histone modifications with high accuracy^33^,^34^, our results suggest that strong chromatin marking is not only associated to high expression levels, but also to high transcription stability across cell types and tissues. This association has been apparently conserved during evolution, since we found that the conservation of the active epigenetic signals is stronger in constrained compared with unconstrained genes (Fig. 5b). These results suggest that constrained and unconstrained genes may be under globally different epigenetic programmes. Further supporting this, we found that constrained genes are regulated by broad promoters (as defined by the FANTOM consortium^6^,^35^) more often than unconstrained genes (67% versus 52%, test of proportions, *P*-value≈0) that their promoters host more transcription factor (TF) chromatin immunoprecipitation sequencing (ChIP-seq) peaks (using data collected in the ENCODE project^7^, Supplementary Methods), than unconstrained genes (Fig. 5c), and are slightly depleted in repeat elements at their transcription start site (density of repeat elements in the promoter region 0.82 versus 0.87, Wilcoxon test, *P*-value≈0.03, Supplementary Methods). Moreover, when performing principal component analysis to classify the promoters of human–mouse orthologous genes based on ChIP-seq measured binding strength of TFs, we found a separation between promoters of constrained and unconstrained genes, although the components capture only a small fraction of the whole variance (Fig. 5d).

**Figure 6:**
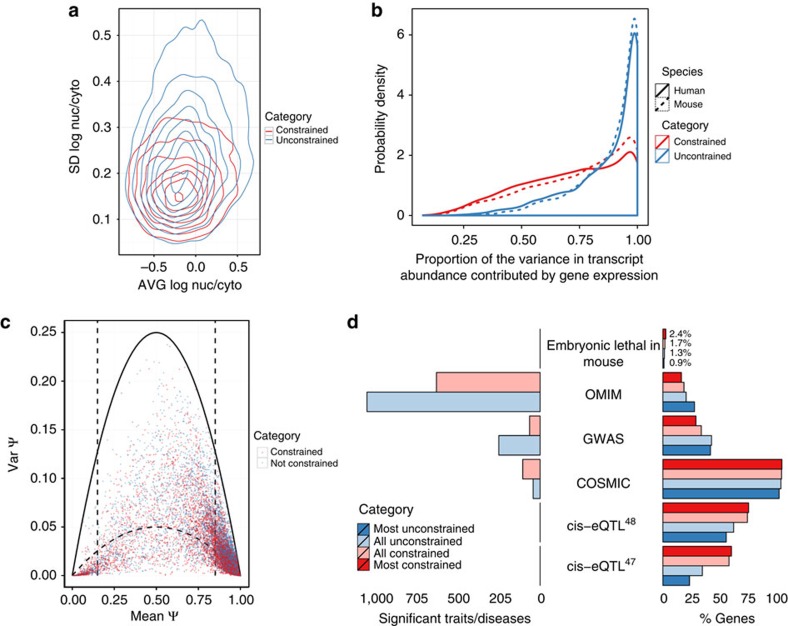
Other properties of constrained genes. (**a**) Contour plots of the joint probability distribution of the average versus standard deviation (s.d.) of log_10_ nuclear-to-cytosolic ratio in constrained and unconstrained genes. (**b**) Distribution of the proportion of the variance in transcript abundance across human and mouse samples that can be explained by the overall variance in gene expression. Values close to 1 indicate that changes in abundance of transcript isoforms originate mostly from changes in gene expression, values close to 0 suggest that most of these changes are due to splicing changes in the relative proportion of transcript isoforms. (**c**) Mean versus variance of splice junction inclusion (*ψ*) in constrained and unconstrained genes. To identify junctions with constrained inclusion at intermediate levels, we set a threshold (inner parabola) of 20% of the maximum variance for the given mean in a Bernoulli distribution (outer parabola) in the interval of mean inclusion (0.15, 0.85). (**d**) The percent of all and of the 1,000 most constrained and unconstrained genes (right) that are lethal in mice according to the Jax mice embryonic lethality database^54^, have hits in OMIM^45^, the NHGRI GWAS catalogue^46^, COSMIC^44^ and that have eQTLs^47^,^48^. On the left, the number of significant traits/diseases associated to constrained and unconstrained genes in OMIM, the NHGRI GWAS catalogue and COSMIC.

The maintenance of constrained RNA production levels appears to have also impacted post-transcriptional regulation. First, genes with constrained gene expression tend to be constrained also in their cellular localization. That is, their ratio of cytosolic versus nuclear abundance across human cell lines is much less variable that for unconstrained genes (Fig. 6a). Moreover, transcripts from constrained genes tend to be enriched in the cytosol compared with transcripts from unconstrained genes (70% of the constrained genes are mostly found in cytosol compared with 60% of unconstrained ones (test of proportions, *P*-value≈0, Supplementary Methods)). Second, we found that splicing plays a comparatively more important role determining the cellular abundance of transcript isoforms in constrained than in unconstrained genes. Gonzalez-Porta *et al.*^36^ developed a method to estimate the fraction of the variance in transcript abundance that can be explained by variance in gene expression, when measured across a number of samples (cell types). Here we found that this fraction is on AVG 0.81 in human cell lines, and 0.82 in mouse tissues—indicating that overall regulation of gene expression plays the major role in defining cell type specificity. This is consistent with tissue-dominated clustering of gene expression profiles compared with the species-dominated clustering of splicing profiles^9^,^10^. We have found, however, substantial differences between constrained and unconstrained genes. The AVG fraction of the variance in transcript abundance explained by gene expression is 0.76 and 0.79 for constrained genes, in human and mouse, respectively, whereas these values are 0.91 for both species for unconstrained genes (Wilcoxon test, *P*-value≈0, Fig. 6b). Thus, regulation through splicing appears to play comparatively a more important role in constrained than unconstrained genes.

Consistent with the relatively minor role of splicing in defining cell type specificity, compared with expression, we found that about 83% of orthologous splice junctions (65,485 out of 78,602 in our set of constrained and unconstrained genes matched by expression) are systematically included at inclusion levels *ψ* larger than 0.85 in all human and mouse samples. We have also found a small set of 123 junctions with extremely low inclusion levels (*ψ*<0.15) in all human and mouse samples. Among the remaining ~20,000 junctions, we used an approach parallel to that for gene expression, to identify junctions, the inclusion of which is constrained across cell types and species. By setting a threshold on the variance of *ψ* to be 20% of the maximum possible variance for a Bernoulli distribution with the given mean, we have identified a set of 1,430 orthologous splice junctions with inclusion constrained at intermediate levels (0.1<*ψ*<0.9, Fig. 6c and Supplementary Fig. 14) across all human and mouse samples. That is, these are junctions with similar inclusion levels in all human and mouse samples investigated here. A majority (59%) of these junctions constrained in splicing belong to genes that are also constrained in expression, consistent with the comparatively more important role of splicing in the regulation of these genes.

## Discussion

‘Housekeeping’ genes are characterized as genes involved in the maintenance of a cell’s basic functioning and are expressed in a ubiquitous and uniform manner in different biological conditions^37^. Numerous collections of housekeeping genes have been proposed^37^,^38^,^39^, but the membership overlap is only moderate (Supplementary Table 8). Here by probing gene expression simultaneously across biological conditions and species—and therefore by introducing an evolutionary component—we found a principled way to identify a set of genes with constrained gene expression. Although not identical, this set of genes has considerable overlap with two of the largest housekeeping gene sets recently reported: 43% of our genes are included in the set of 3,664 housekeeping genes reported by Eisenberg and Levanon^37^, and 69% in the set of 6,560 stably expressed genes that we have derived from the data reported by FANTOM5 (F5)^6^ (Supplementary Methods and Supplementary Fig. 15).

Here we have found that genes with evolutionary constrained levels of expression account for the bulk of transcription in differentiated mammalian cells, meaning that a substantial fraction of the RNA content in mammalian cells remains remarkably constant across large biological and evolutionary scales. Although the evolutionary forces responsible for this transcriptional stability have not left an obvious imprint in the sequence of mammalian genomes^29^, we have found, in contrast, a remarkable epigenetic signature. Our results show that the evolutionary stability in gene expression is associated with strong and consistent marking by active histone modifications. This indicates that chromatin marking is not only a reflection of transcriptional levels, but also of transcriptional stability. This marking, moreover, is conserved across the evolutionary distance separating human and mouse. Genes with constrained expression seem to have evolved, in addition, a characteristic programme of post-transcriptional regulation, in which sub-cellular localization and alternative splicing play a more prominent regulatory role than in genes with unconstrained expression. These results are consistent with an evolutionary interplay between transcriptional regulation and regulation by splicing, in which the maintenance of tight expression levels would enhance the role of splicing as a mechanism to modulate the abundance of individual transcript isoforms. They also suggest that lack of sequence constraint (which is detectable over less than 10% of the mammalian genomes, according to recent estimates^40^) cannot be naively equated to lack of biological function^41^.

The essential role of genes with constrained expression in basic cellular architecture and function suggests that disruption of their functions is likely to have dramatic phenotypic consequences, including cell survival. Although the relationship between housekeeping genes and disease remains controversial^42^,^43^, we indeed found that mutations in genes with constrained levels of expression are most often associated with embryonic lethality, and that somatic mutations in these genes are associated to a broad spectrum of cancerous phenotypes^44^ (Fig. 6d). In contrast, mutations in unconstrained genes are more likely to be associated with other diseases (as reported in OMIM^45^ and the NHGRI GWAS catalog^46^, Fig. 6d), as expected, as mutations lethal during development are unlikely to cause detectable diseases. The larger number of GWAS hits associated to unconstrained genes underscores the complexities involved in identifying disease causative genetic variations when these affect gene region. We found that the expression of unconstrained genes is also more variable across individuals compared with the expression of constrained genes (Supplementary Fig. 16). Large expression variability decreases the power to identify eQTLs, and indeed we observed that unconstrained genes, which are more often associated to diseases, are substantially depleted for eQTLs compared with genes with constrained levels of expression^47^,^48^ (Fig. 6d).

In summary, by introducing an evolutionary dimension, we identified in a principled way, a set of genes with constrained expression across cell types and species. These genes contribute to a core invariable component of mammalian transcriptomes, contributing thus little to cell type specificity and species differentiation. They are the main drivers of many transcriptional features that have been reported as conserved across mammalian transcriptomes.

## Methods

### Genomes and annotation sets

Throughout this work we used February 2009 assembly of the human genome (hg19,GRCh37) and July 2007 assembly of the mouse genome (mm9, GRCm37)^49^. Human Gencode v10 and mouse ENSEMBL v65 databases were used for transcript annotations. Genomes were partitioned into exonic, intronic, genic and intergenic regions as explained in Supplementary Methods.

### Mouse tissue acquisition

C57Bl/6 mice were used for all tissue resections. The following tissues were taken from 8-week-old littermates: adrenal, duodenum, stomach, genital fat pad, subcutaneous fat pad, large intestine, small intestine, ovary, testis, spleen, colon, lung, heart, kidney, liver, thymus, mammary gland, placenta (from pregnant mice), cortex, frontal lobe, cerebellum, bladder, liver (Supplementary Table 1). Central nervous system (CNS) was taken from stage E11.5 littermates. CNS and liver were taken from E14 littermates. Liver, limb and whole brain were taken from E14.5 littermates. CNS and liver were taken from E18 littermates. All animal studies were conducted with approval from the Institutional Animal Care and Use Committee.

### Human cell culture and RNA isolation

Cells were grown according to the ENCODE cell culture standards. RNA was prepared as was described previously^8^.

### Library construction

We generated directional (stranded) libraries for paired end sequencing on the Illumina platform as described in ref. 50 and as also used in ref. 8 to generate the human ENCODE data. Briefly, 100 ng of Ribominus (Invitrogen Inc.)-treated PolyA(+) RNA with length >200 nt were mixed with 2 ng of exogenous RNA spike-in, pool 14 (ref. 51). A mixture of random hexamers and oligo-dT_21_ were used to prime the reverse transcriptase reaction. Entry sites for second strand synthesis catalysed by *Escherichia coli* DNA Polymerase are generated through RNAse H nicks of the DNA:RNA duplex. dTTP is replaced with dUTP during the second strand synthesis. The (double stranded) complementary DNA is then sheared using sonification (Covaris). Staggered ends generated during shearing, are repaired and adenylated to prime them for adapter ligation with Illumina Y-adapters. The second strand containing dUTP is eliminated using UNG digestion. The resulting (single stranded) complementary DNA is run on an agarose gel and bands with the desired insert sizes of ~200 nt are cut out. Cluster compatible sequences are appended in an 18-cycle PCR reaction and the final library is gel purified. All libraries from biological replicates (littermates) were prepared in parallel to minimize day‐to‐day variation in the experimental procedure. Finally, the libraries were sequenced on the Illumina GAIIx or Hi-Seq platform to an AVG depth of ~100 million mate pairs per sample. The human ENCODE RNA-seq data are at GEO: GSE30567. The mouse ENCODE RNA-seq data are at GEO: GSE36025 (Supplementary Table 1).

### RNA-seq experiments

Stranded paired-end RNA-seq data from 18 human cell lines and 30 mouse tissues and developmental stages in two bio-replicates were generated as described previously^3^. The raw data (FASTQ), mapped data (BAM) and lists of quantified elements are available at http://mouse.encodedcc.org/. These data, as well as additional data on all intermediate processing steps, are also available on the RNA Dashboard ( http://genome.crg.cat/encode_RNA_dashboard/).

### RNA-seq data processing

RNA-seq reads were aligned to the human (hg19) and mouse (mm9) genomes using the STAR 1.9 software^52^ with up to ten mismatches per paired alignment and without using the annotations. Only alignments for reads mapping to ten or fewer loci were reported. Mapped reads were used to generate contigs, splice junctions and *de novo* transcript models. Non-parametric irreproducibility ascertainment (npIDR) was used for genomic elements (such as splice junctions, exons, transcripts, etc) by requiring npIDR≤0.1. Strand-specific contigs were called from merged biological replicates independently of the annotations and assessed with npIDR. Cufflinks 1.0.3 (ref. 16) was used to assemble *de novo* transcripts from STAR alignments considering only uniquely mapping non-duplicated alignments crossing GU/AG junctions. The alignments from the two bio-replicates were merged before Cufflinks assembly. Cufflinks transcript models passing the npIDR threshold (npIDR≤0.1) were pooled across all experiments and further filtered by requiring CAGE data support in FANTOM study^6^. The Cufflinks gene, transcript and exon RPKM were quantified using Flux Capacitor^53^ in each bio-replicate, and the resulting RPKM were assessed for reproducibility using npIDR^8^. AVG read density was computed by using only uniquely mapped reads, separately for each bio-replicate and for each strand without applying npIDR. The quantitative assessment of splicing at the level of splice junctions was done by using intron-centric metrics^25^. More details on RNA-seq data processing are in Supplementary Methods.

### Gene expression quantification and analysis

Transcript RPKMs were quantified using Flux Capacitor^53^ with respect to the subsets of GENCODE v10 and ENSEMBL v65 annotations corresponding to long transcripts (Supplementary Table 2). Gene (respectively, exon) RPKMs were computed as sums of RPKMs of all their annotated transcripts (respectively, transcripts that contain the given exon). The base-10 logarithm of the AVG gene RPKM, taken across samples, was chosen to be the gene expression level after assessing reproducibility between bio-replicates with the non-parametric version of IDR (IDR≤0.1); non-reproducible RPKM values were set to zero^8^. A two-factor analysis of log expression levels versus gene and condition was performed by the standard ANOVA package in R. Zero RPKM values were replaced by the effective value of 10^−3^. The computation of variance was performed separately for human and mouse data matrices, which were constrained to the lists of orthologous genes. The normality of the data was not required as we did not carry out significance tests related to ANOVA.

### Quantification of histone modification levels

Whole-genome human and mouse ChIP-seq profiles were analysed for GEO accession numbers listed in Supplementary Table 7. Only data without additional chemical treatment was considered. In order to construct histone mark profiles, the read density was averaged across a group of genes (for example, constrained versus unconstrained; see below) for each nucleotide in the 1-kb window around the transcription start site for H3K4me3 and H3K27me3 marks or the TTS for H3K36me3. The AVG signal across a group of genes was normalized to the maximum signal across all genes for each mark, and then it was averaged across samples in each species. The comparison of histone marks between species is described in Supplementary Methods.

### Dynamic range

The DNR of a mouse/human one-to-one orthologue is computed based on the non-zero RPKM of the gene in each experiment of this species once npIDR has been applied, and is only defined for genes with non-zero expression in at least two mouse experiments and at least two human experiments. For such genes, the DNR is computed as the difference between the base-10 logarithm of the maximum RPKM of the gene in all experiments in all species, and the base-10 logarithm of the minimum RPKM of the gene in all experiments in all species. A DNR value can be assigned to 14,363 mouse/human one-to-one orthologues, out of the total 15,736 gene orthologues.

### Constrained genes

Genes with constrained expression, shortly referred to as *constrained genes*, are defined as genes with DNR less or equal to 2 across all mouse and human experiments (Supplementary Table 1). Unconstrained genes are the genes with a defined DNR (that is, ones with at least two non-zero expression levels in each species) but that are not constrained. Out of the 14,363 genes with a defined DNR, 6,636 are constrained and 7,727 are unconstrained (Fig. 3b). Additional protocols on the construction of constrained and unconstrained gene sets are explained in Supplementary Methods.

### Vls/Vt

Gene expression contribution to the transcript abundance variation was computed following the methodology presented in ref. 36. In a nutshell, for each gene, samples are represented in a multidimensional space using their transcript abundances as coordinates. The contribution of gene expression in the transcript abundance variation is computed by the variation after projecting the samples into a model of constant splicing (a line in the multidimensional space) divided by the total variation without projection. If this ratio is close to 1, projecting into the ‘no splicing’ model did not reduce the transcript variation, pointing at mainly gene expression contribution. Inversely, if close to 0, alternative splicing is mostly responsible for the major part of the transcript variation. In addition, we implemented two improvements on the version described in ref. 36. First, the effect of outlier samples is mitigated through a bootstrapping approach. Second, overestimation of gene expression in situations with extreme major isoform was reduced by rescaling transcript abundances using square-root transformation.

## Author contributions

T.R.G., R.G., and C.A.D. designed and managed the project; L.-H.S. prepared biological samples; L.-H.S. and M.F. prepared RNA samples; L.-H.S., J.D. and M.F. prepared Illumina RNA-seq libraries; C.Z. and J.L. administered computer infrastructure and quality control for data storage and analysis; D.D.P., S.D., A.B., A.D., P.P.B., G.B., B.P., S.B., J.M., A.T., A.H., M.A.B., C.N. analysed data; M.G. and M.A.B. assisted with manuscript preparation, T.R.G., R.G., D.D.P., S.D. and A.B. wrote the paper with input from all authors. All authors discussed the results and commented on the manuscript.

## Additional information

**How to cite this article:** Pervouchine, D. D. *et al.* Enhanced transcriptome maps from multiple mouse tissues reveal evolutionary constraint in gene expression. *Nat. Commun.* 6:5903 doi: 10.1038/ncomms6903 (2015).

**Accession codes.** RNA-seq data have been deposited in Gene Expression Omnibus under the accession codes GSM900183-GSM900199, GSM1000562-GSM1000574, GSM981256, GSM981249, GSM984609 and GSM981253 (GEO: GSE30567 and GEO: GSE36025).

## Supplementary Material

Supplementary Information Supplementary Figures 1-16, Supplementary Tables 1-8, Supplementary Methods and Supplementary References
