# Supplementary material for: Enhanced transcriptome maps from multiple mouse tissues reveal evolutionary constraint in gene expression
Source: Nat Commun. 2015 Jan 13;6:5903. doi: 10.1038/ncomms6903 (PMC4308717; doi:10.1038/ncomms6903)
Supplement: Supplementary Information — Supplementary Figures 1-16, Supplementary Tables 1-8, Supplementary Methods and Supplementary References [file ncomms6903-s1.pdf]

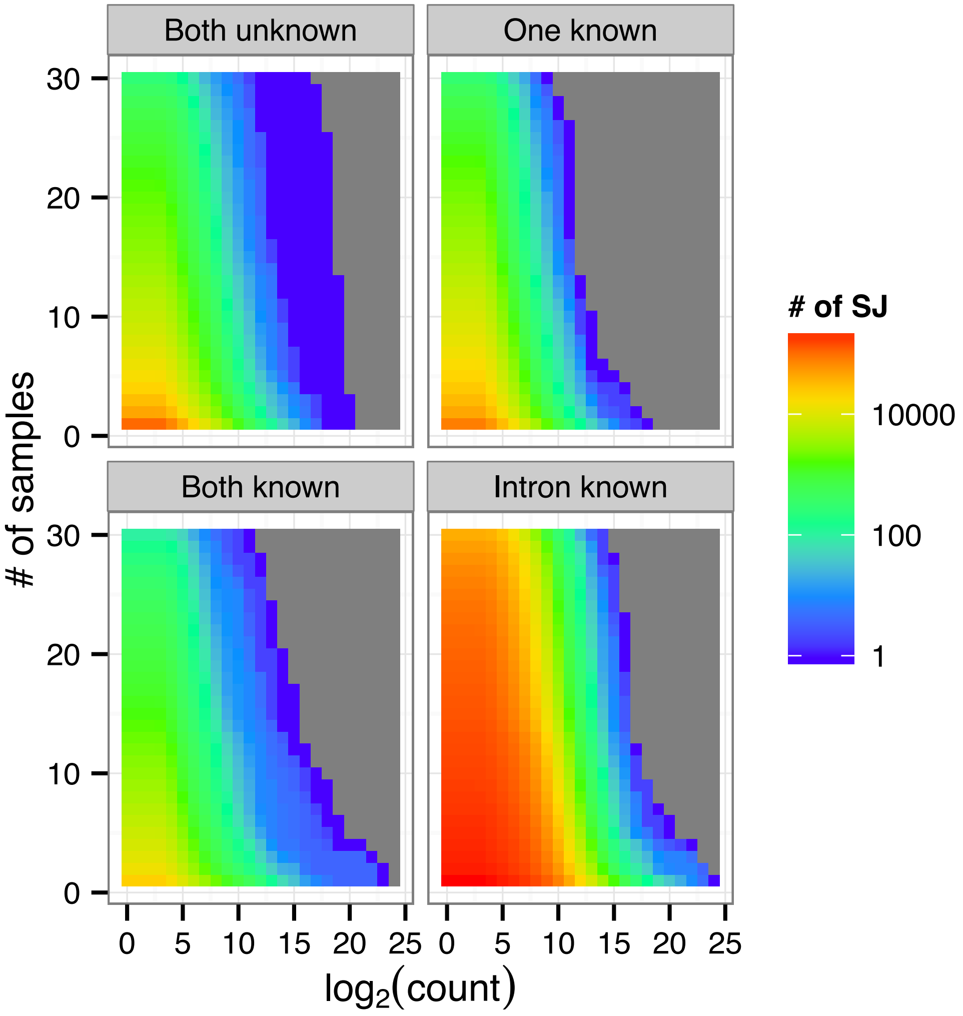

Supplementary Figure 1: The distribution of counts of splice junctions in mouse samples. The color at the point (x, y) represents the number of splice junctions that have $\log_2$ count of at least x in at least y samples. The x-axis is $\log_2$ counts; the y-axis is the number of samples.

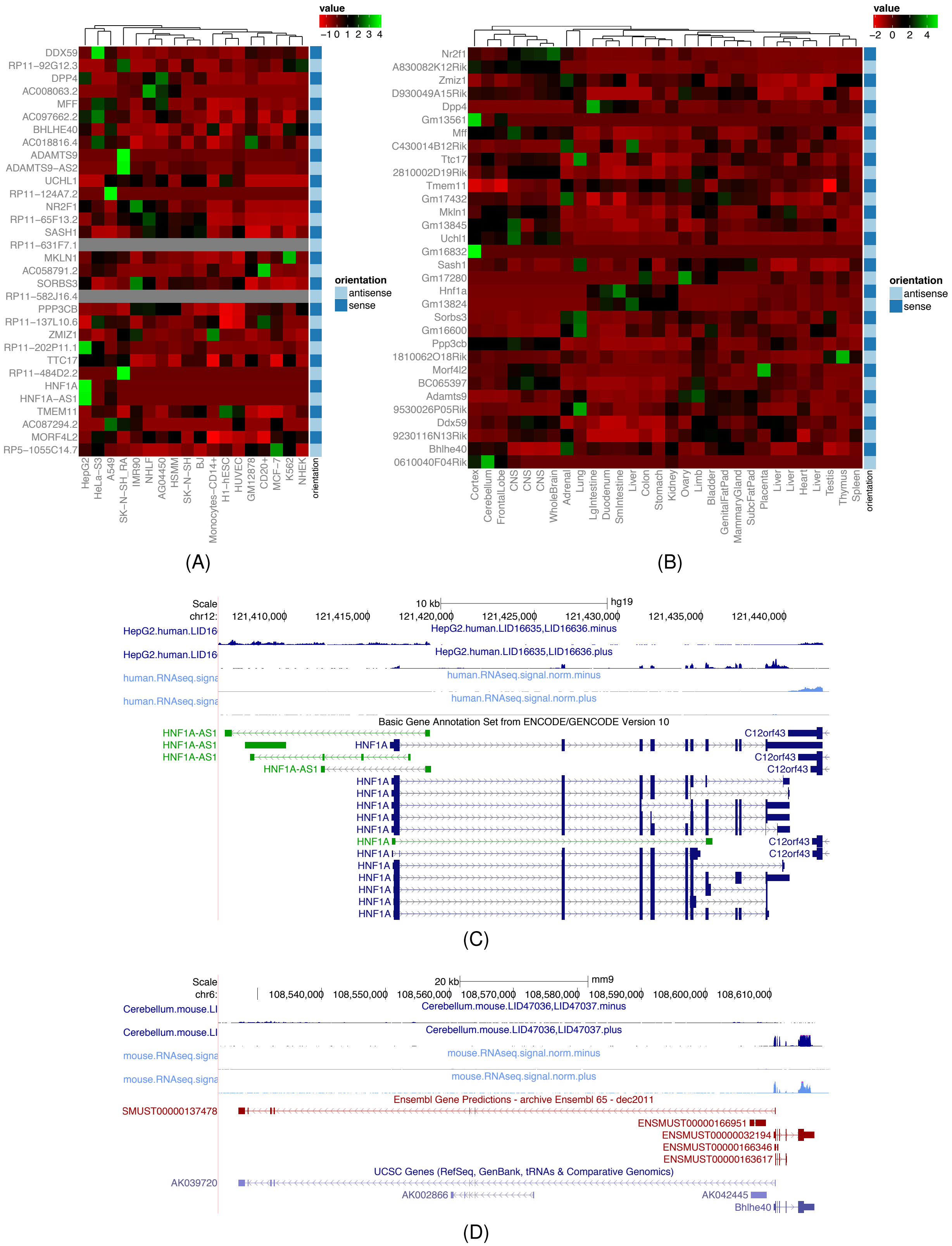

Supplementary Figure 2: Expression of sense/antisense gene pairs. **(A,B)** Heatmaps showing the scaled RPKM of the 16 sense/antisense gene pairs across human (A) and mouse (B) samples. **(C)** The UCSC genome browser screenshot of HNF1A and its antisense in human, showing the tissue-specific expression of this pair in HepG2.

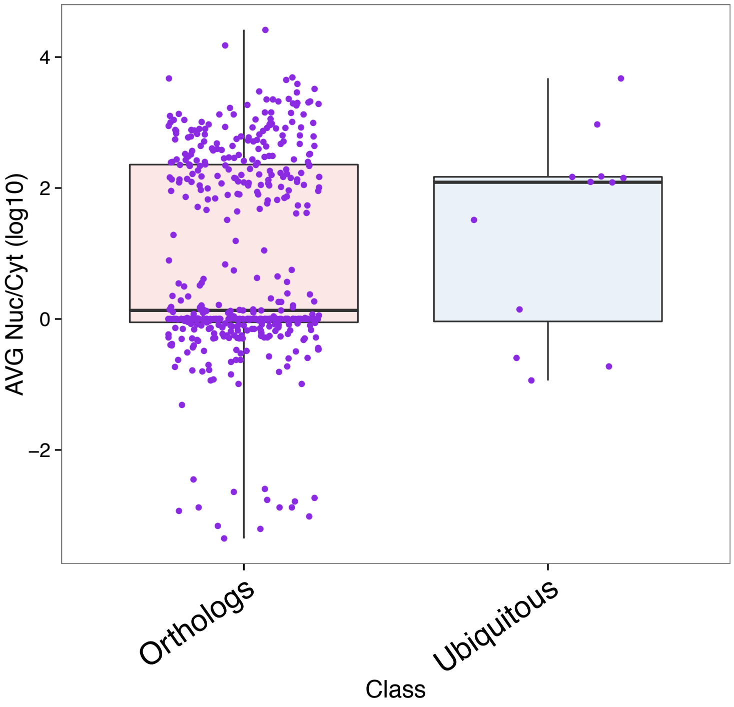

(A)

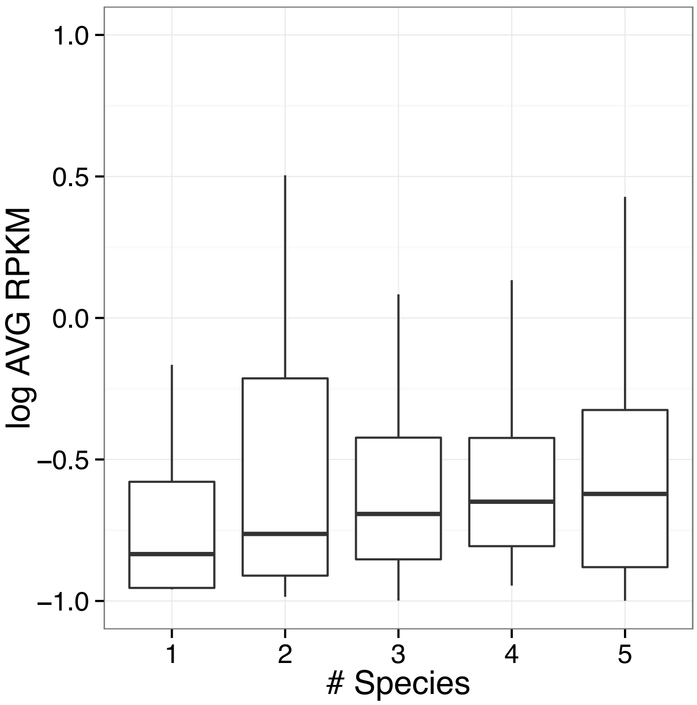

(B)

Supplementary Figure 3: **(A)** Average nuclear versus cytosolic ratio across 7 human cell lines of two sets of 1-to-1 orthologous RNA genes: all (left, median=0) and ones that are expressed in more than 50% of the mouse and 50% of the human samples (right, median=2). **(B)** Average gene expression level (RPKM, across mouse tissues) of mouse 1-to-1 orthologous lncRNAs categorized by the number of species (Mouse, Cow, Pig, Rat, and Dog) in which lncRNA orthologs were detected (threshold for detection is $RPKM \geq 0.1$).

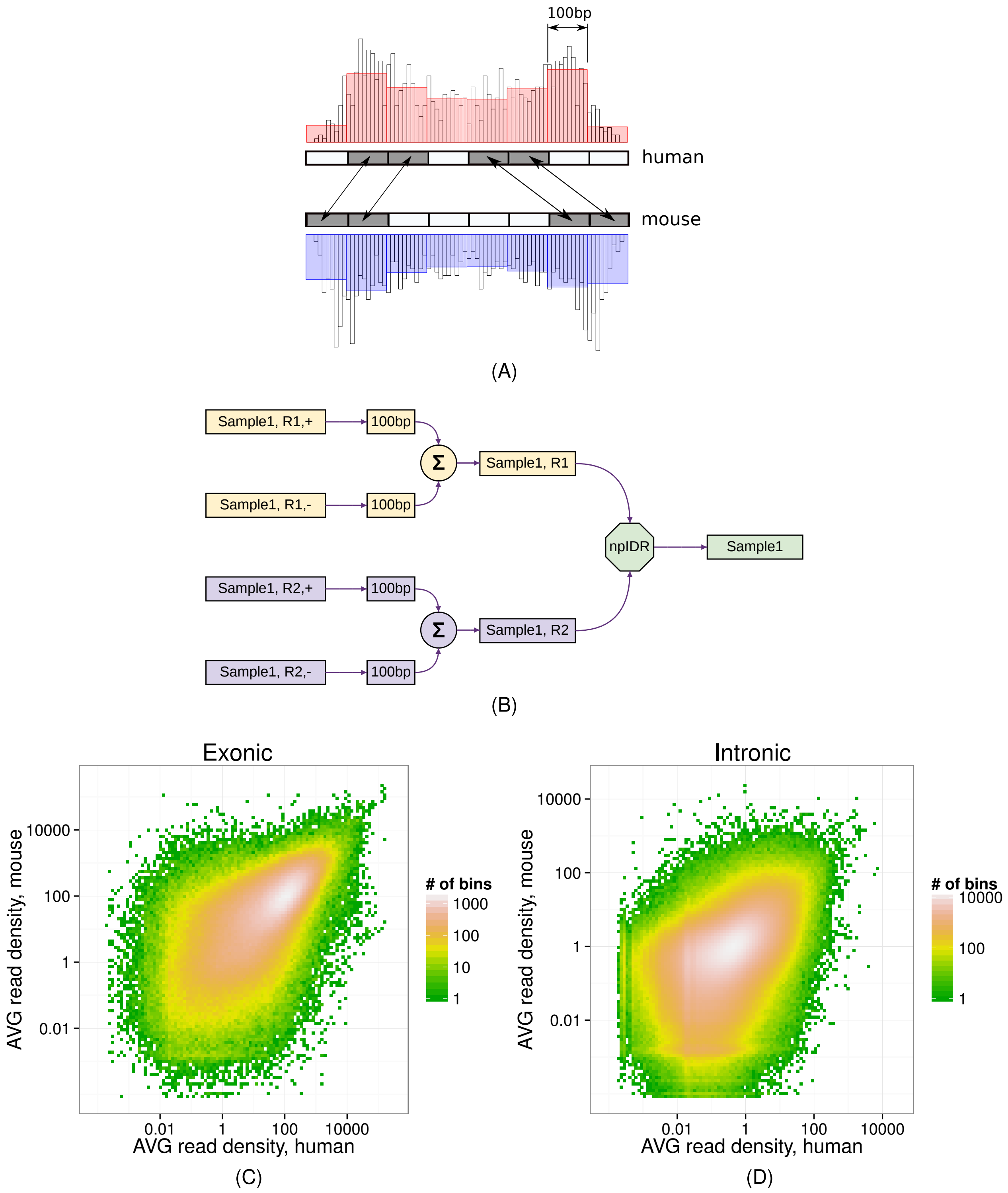

Supplementary Figure 4: **(A)** Nucleotide coverage statistics were averaged over 100-nt bins spaced equally along each genome. The midpoint of each 100-nt bin in human genome was mapped to the mouse genome based on whole genome alignments, inducing the correspondence between bins. **(B)** The flowchart of genome-wide nucleotide coverage processing. **(C,D)** The joint distribution of $\log_{10}$ average read density in orthologous exonic (C, $cc = 0.62$) and intronic (D, $cc = 0.47$) 100-nt bins in human (x-axis) and in mouse (y-axis). Exonic/intronic segmentation is with respect to the human genome.

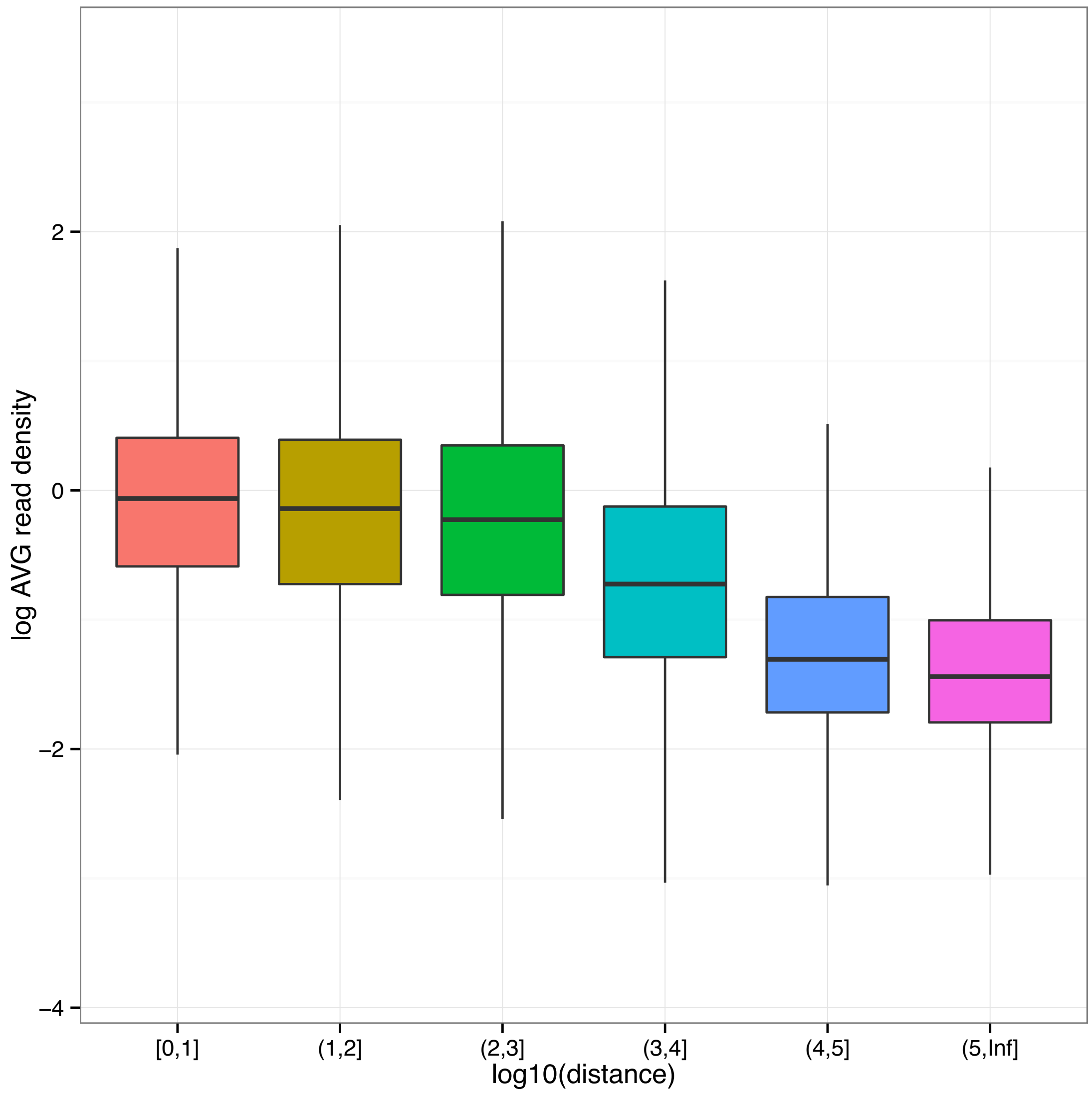

Supplementary Figure 5: The distribution of $\log_{10}$ average read density in 100-nt bins in the human genome as a function of distance from the bin to the nearest gene.

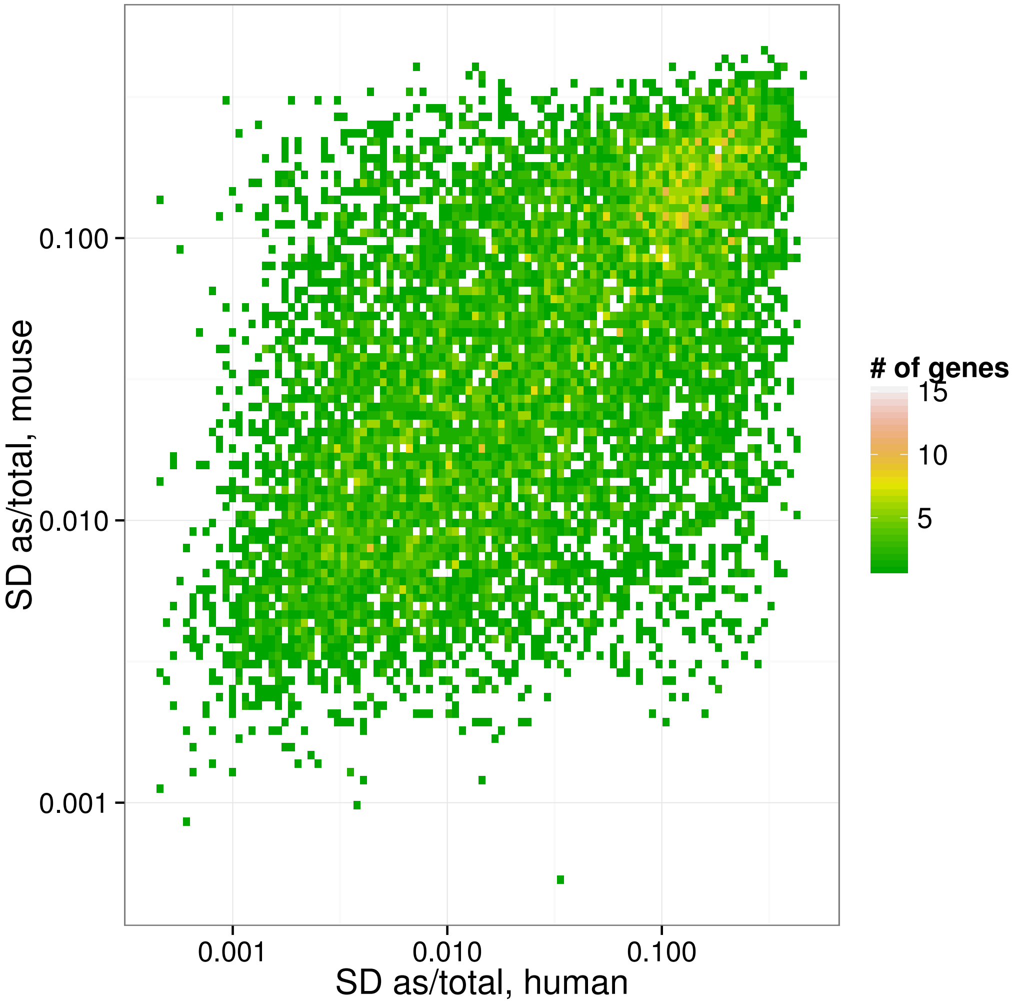

Supplementary Figure 6: The joint distribution of $\log_{10}$ standard deviation (SD) of antisense-to-total ratio in pairs of orthologous genes ($cc = 0.52$). Observations with constant as/total ratio (i.e., with $SD = 0$) were not included.

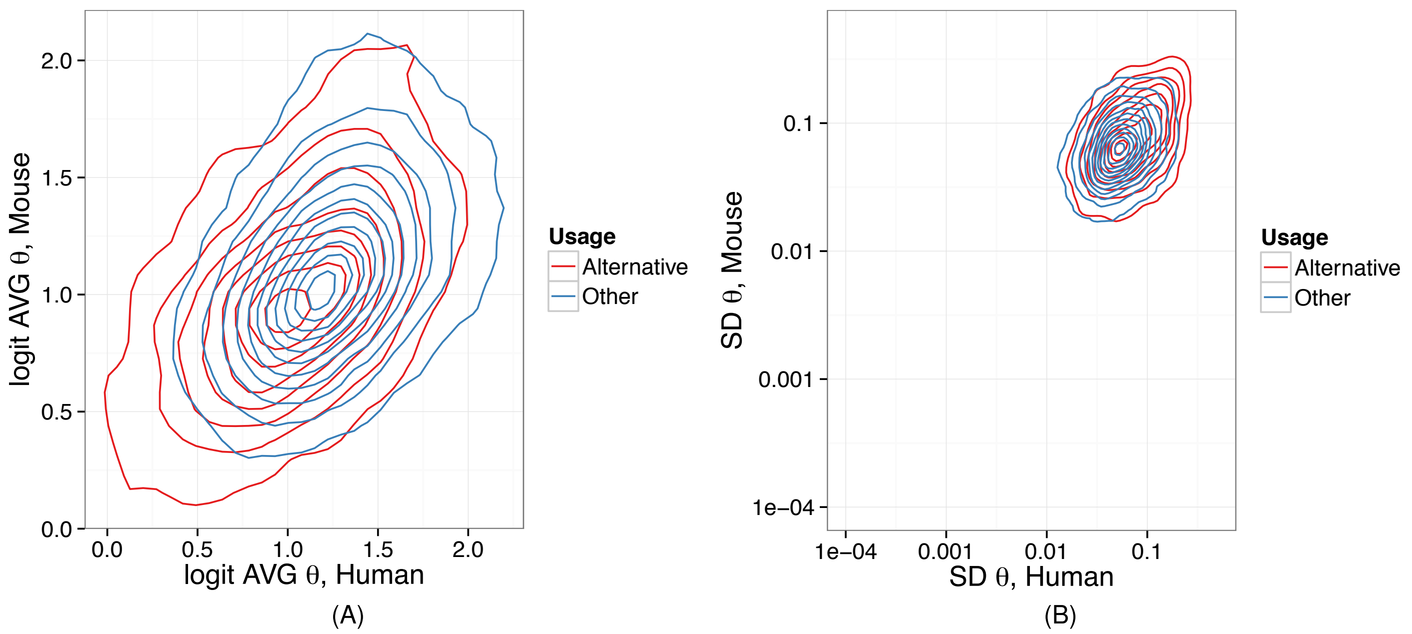

Supplementary Figure 7: **(A)** Contour plots of the joint distribution of average SJ processivity (AVG θ, completeness of splicing index) in pairs of orthologous SJs. "Alternative" denotes SJs that are annotated as alternative in both species. The logistic transformation ($logit$) was applied to AVG θ before computing the probability density. SJ with constant complete inclusion or exclusion are not shown. **(B)** The joint distribution of standard deviation of SJ processivity.

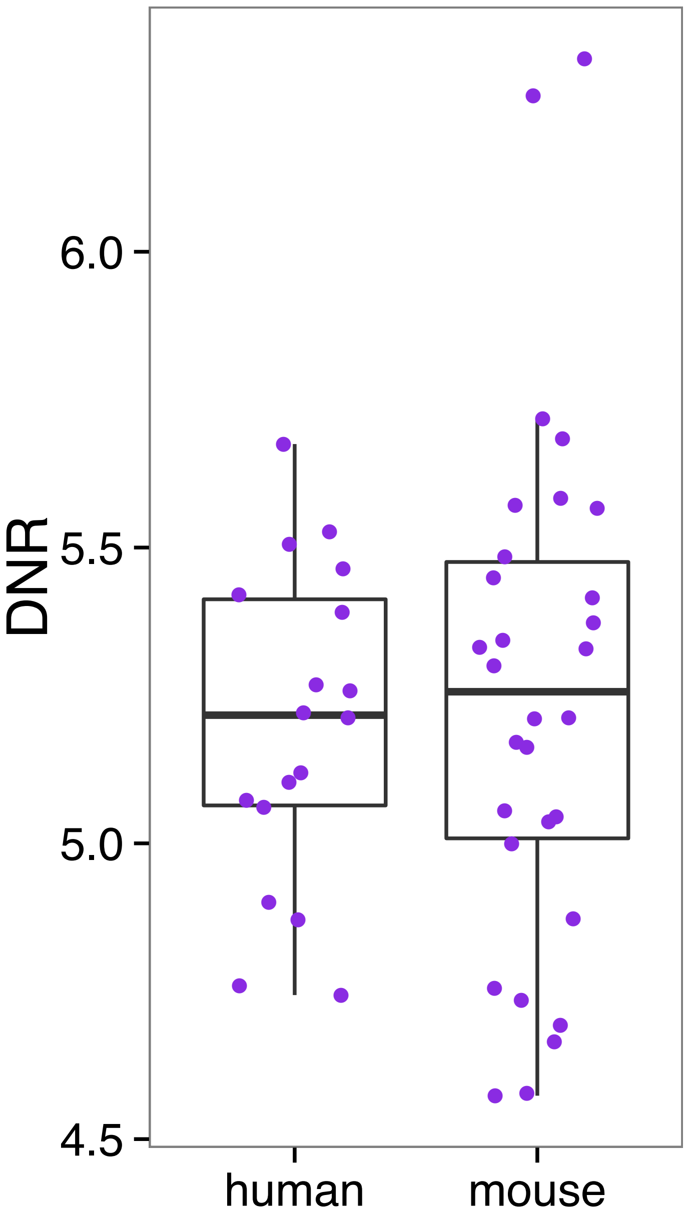

Supplementary Figure 8: Sample dynamic range (i.e., the dynamic range of gene expression within each sample) in human and mouse. The sample dynamic range is computed as $\log_{10}$ of the RPKM of the gene with the largest RPKM minus $\log_{10}$ of the RPKM of the gene with the lowest RPKM. Each dot represents one sample.

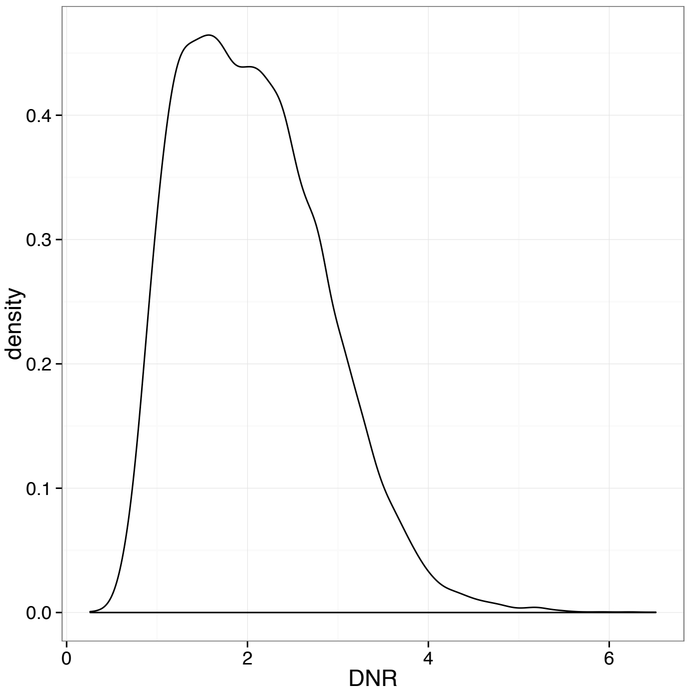

Supplementary Figure 9: The distribution of the dynamic range (DNR, $\log_{10}$ of the ratio of the largest and the lowest non-zero observations) of gene expression level in orthologous genes across human HBM and mouse CSHL tissue samples.

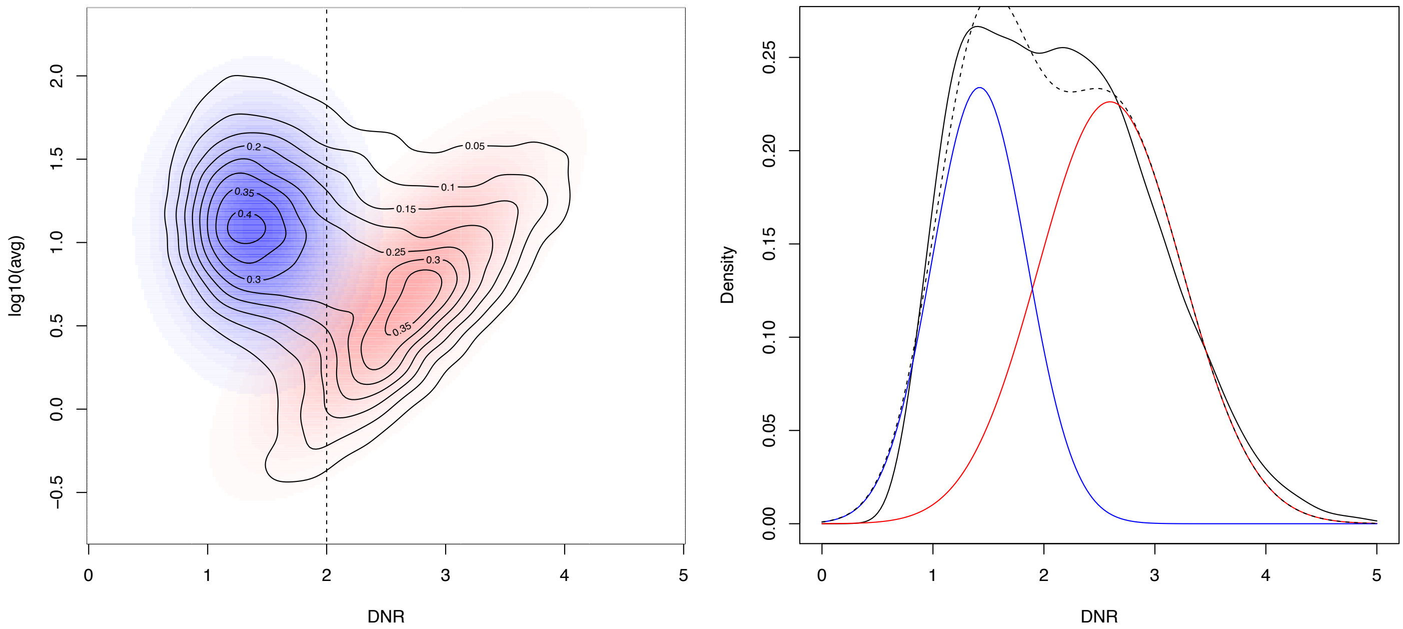

(A) (B)

Supplementary Figure 10: **(A)** The joint probability distribution of the dynamic range (DNR, x-axis) and $\log_{10}$ average gene expression level (y-axis) in protein-coding ortholog genes. The contour plot is in the units of probability density. The joint distribution is approximated by a mixture of two 2D Gaussian components shown in red and blue (see Supplementary Methods). **(B)** The probability distribution of the dynamic range (the marginal distribution) approximated by the sum of the two marginal distributions of the respective components in panel A. The weighted sum is shown by the dashed line.

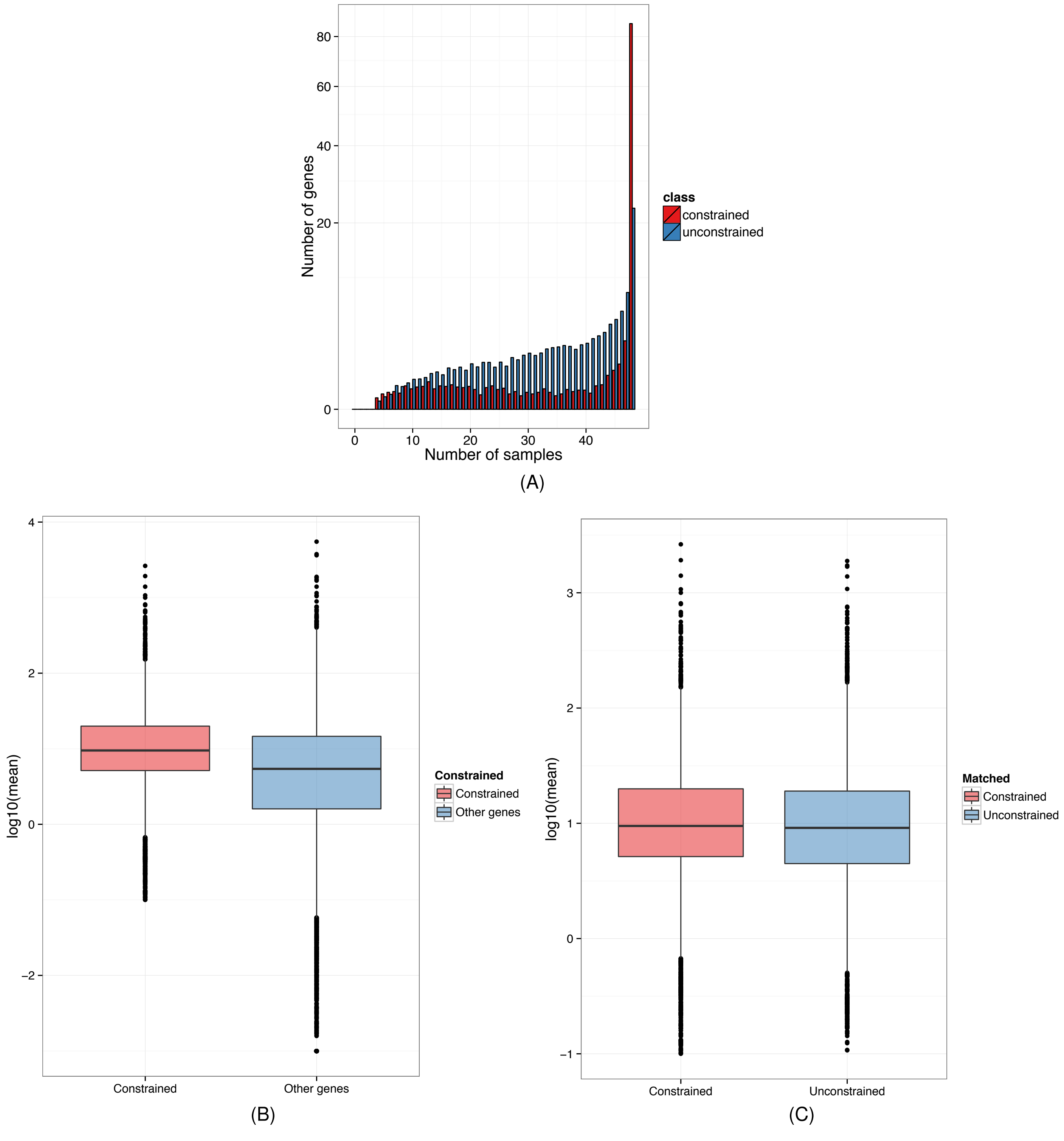

Supplementary Figure 11: **(A)** The distribution of the number of (human and mouse) samples, in which a gene is expressed, for constrained and unconstrained genes. **(B)** The distribution of $\log_{10}$ average gene expression levels (across human and mouse samples) in constrained genes as opposed to that in the rest of orthologous genes. **(C)** The distribution of $\log_{10}$ average gene expression levels in the set of constrained genes and in the set of unconstrained genes with matched expression (see Supplementary Methods).

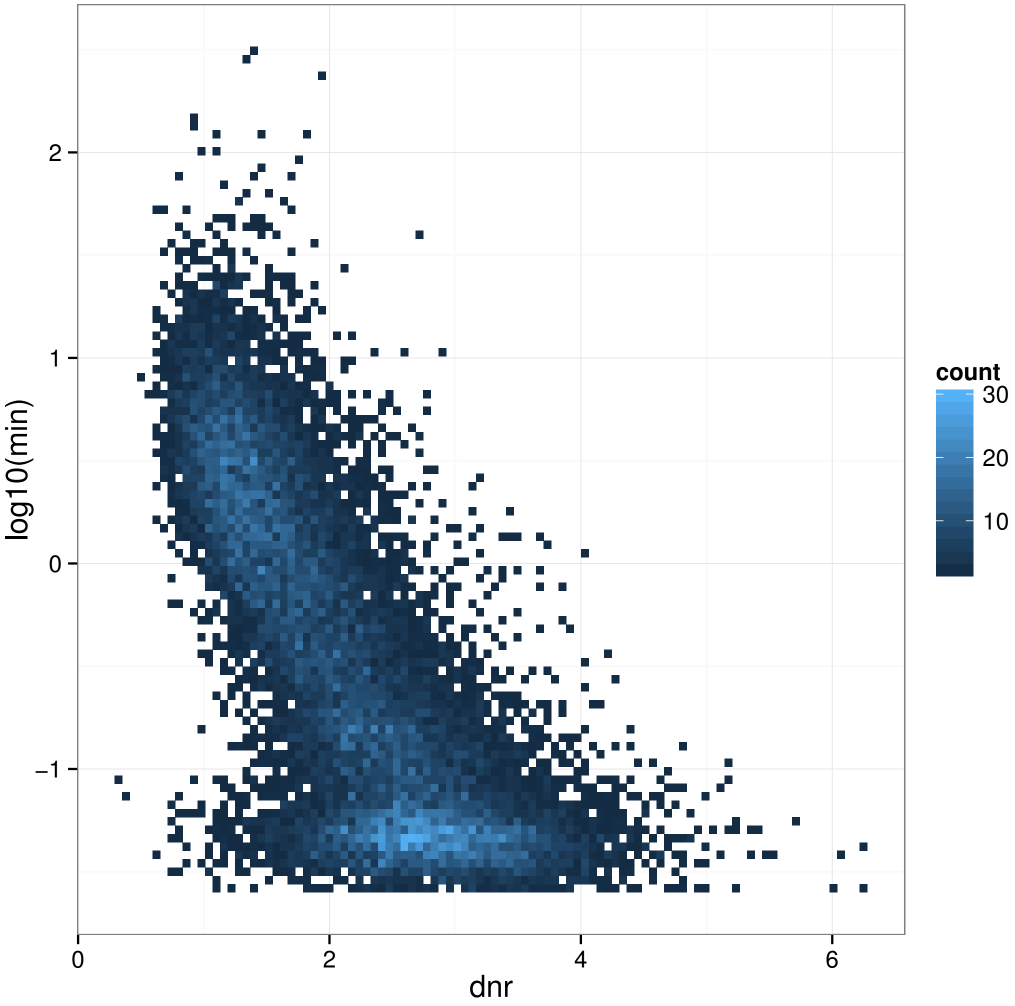

Supplementary Figure 12: The joint distribution of the dynamic range (DNR) vs. $\log_{10}$ of minimum gene expression for 1-to-1 orthologous genes across human and mouse samples.

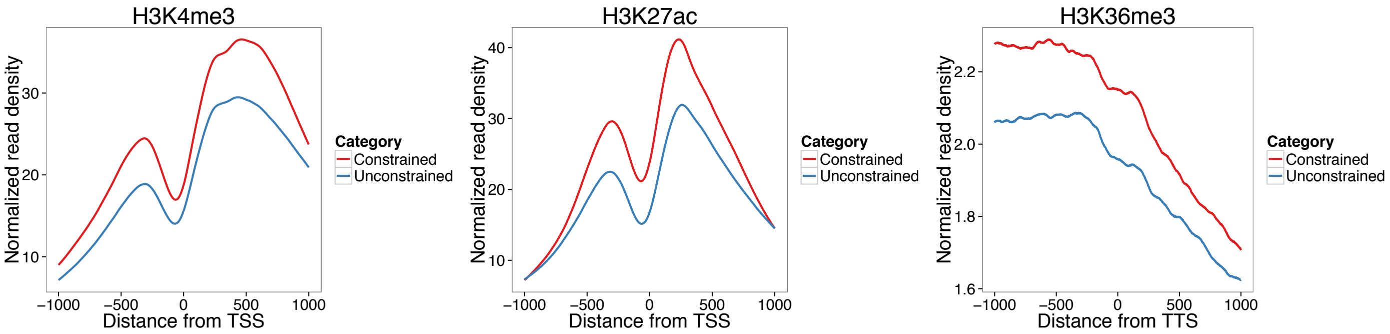

(A) human

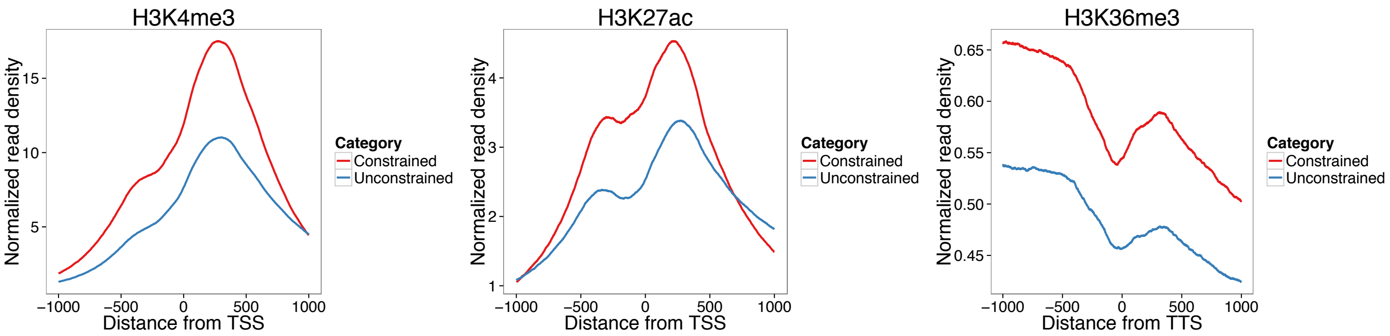

(B) mouse

Supplementary Figure 13: **(A,B)** Histone marking for constrained and unconstrained genes in human HeLa-S3 cell line (A) and adult mouse kidney (B). Shown are the normalized read densities of H3K4me3 and H3K27ac at TSS and the normalized read density of H3K36me3 at TTS, plotted separately for the set of constrained genes (red) and for the set of unconstrained genes with matched expression levels (blue).

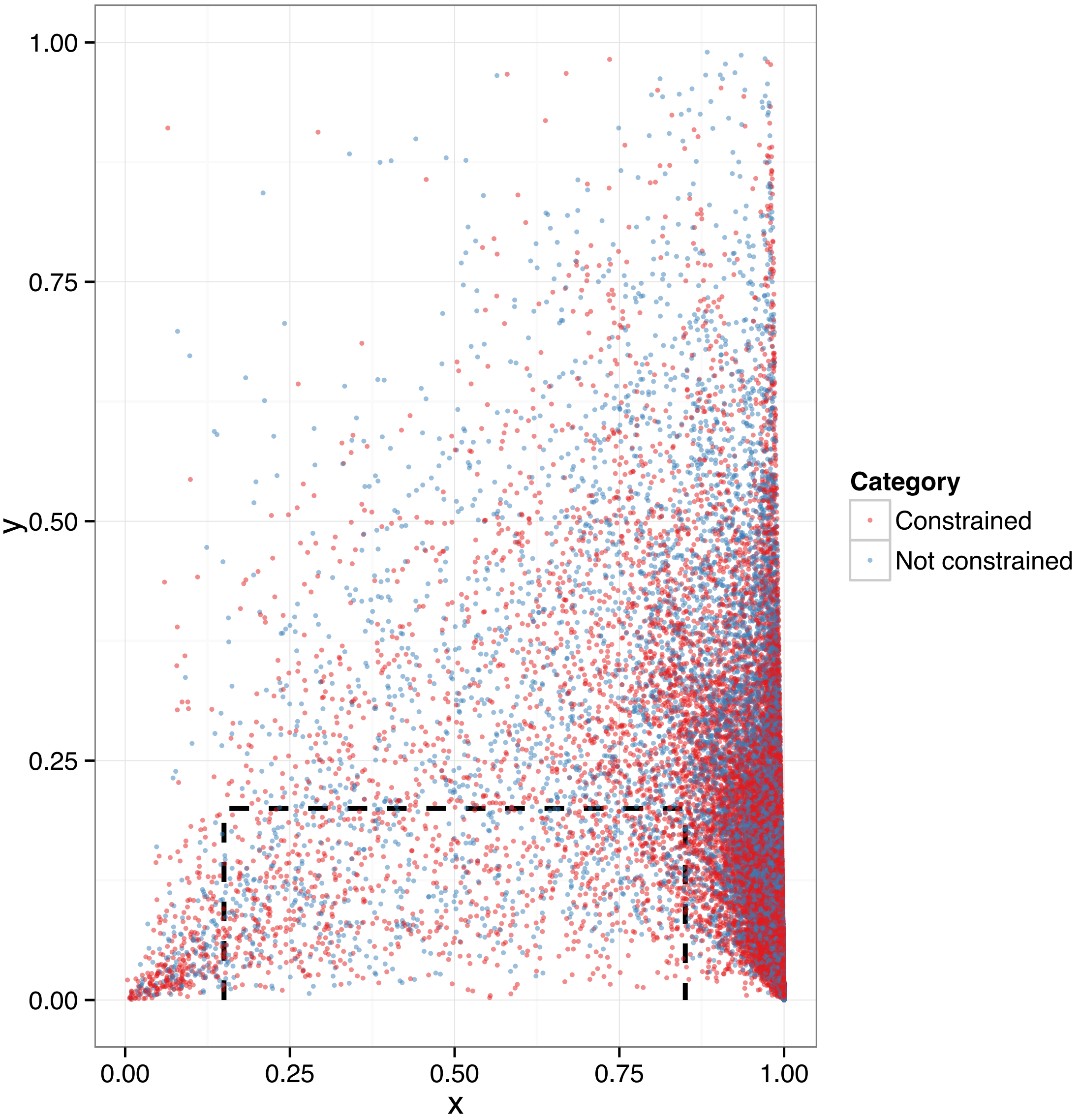

Supplementary Figure 14: Scatterplot of mean vs. fraction of variance of splice junction (SJ) inclusion rate, Ψ, (relative to the maximum possible variance for the Bernoulli distribution with the given mean) for orthologous splice junctions. SJ that belong to constrained (unconstrained) genes are shown in red (blue). SJs with (0.15$< mean(\Psi) < 0.85$) and with relative variance below 20% are delimited by the dashed lines, and are considered SJs with constrained expression at intermediate levels of inclusion.

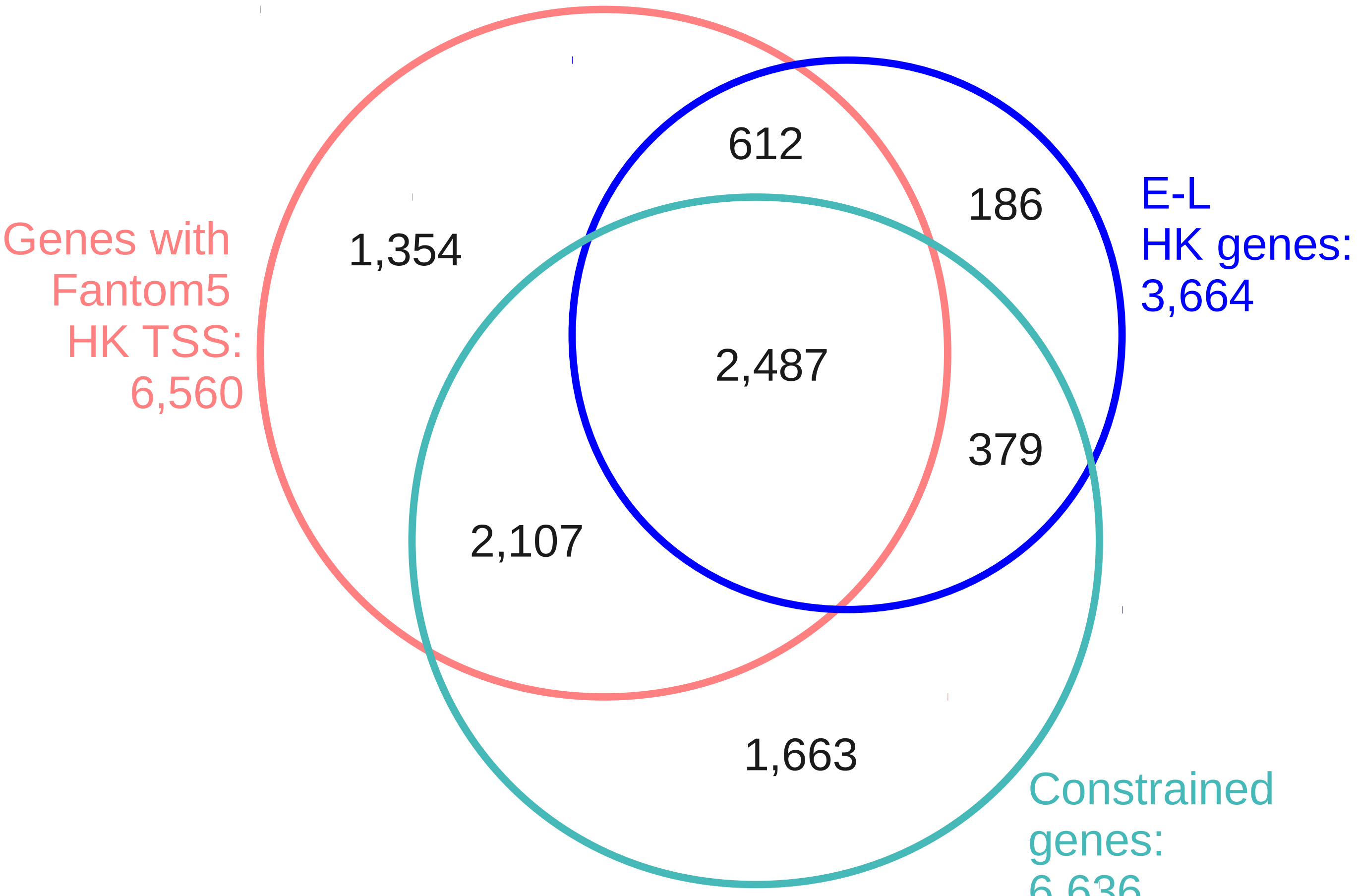

Supplementary Figure 15: The overlap between the set of housekeeping genes derived by deep sequencing from FANTOM 5[4] and by Eisenberg and Levanon (E-L)[25], and the set of genes with constrained expression derived here.

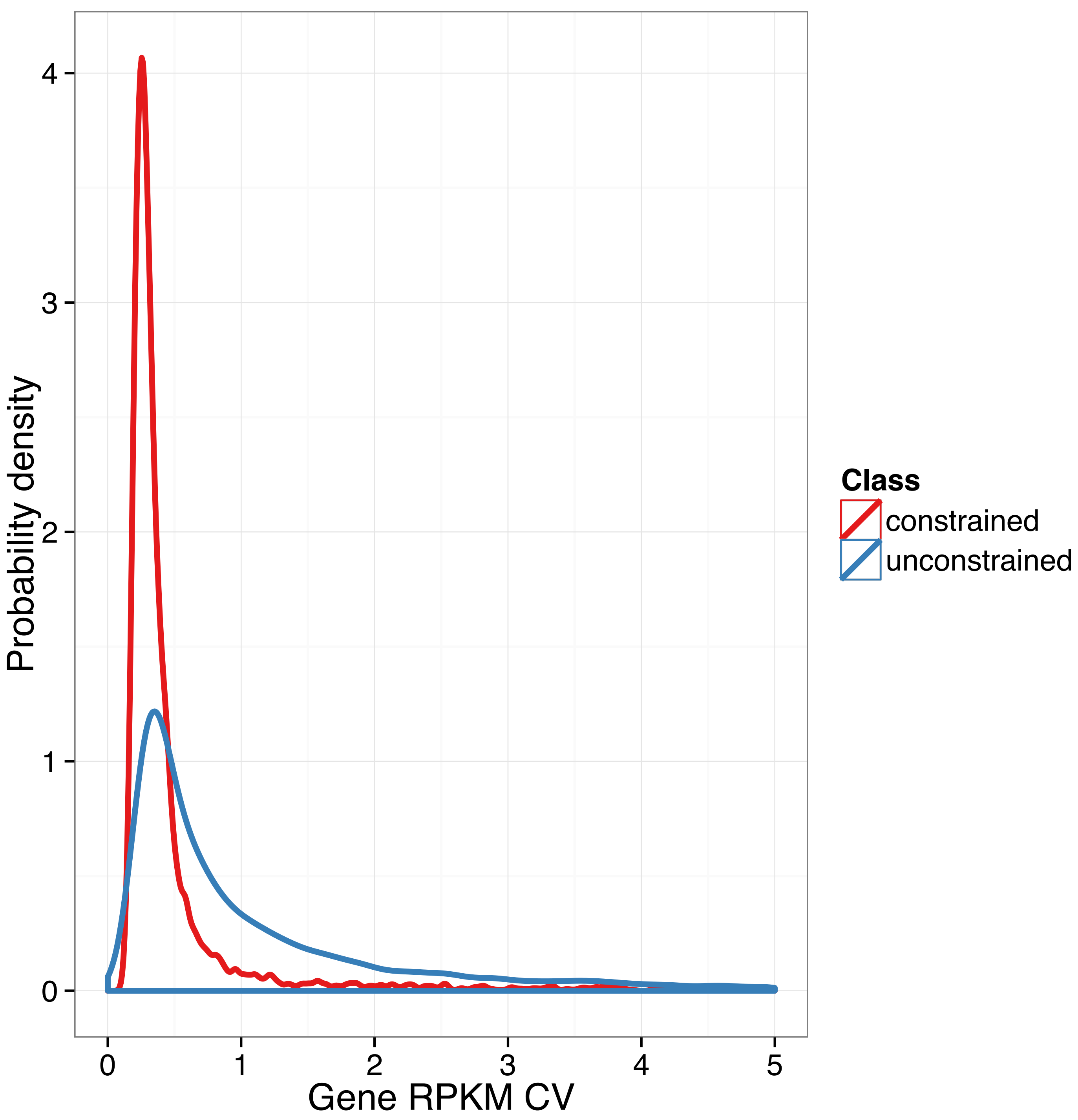

Supplementary Figure 16: The distribution of the coefficient of variation in gene expression in 766 human European samples[33] in constrained and unconstrained genes (see Supplementary Methods for details).

Supplementary Table 1: **Summary of RNA-seq experiments.** Experiments were performed on whole cell polyA+ RNA samples in 18 human cell lines and in 30 mouse samples (25 tissues and 5 developmental stages); two bio-replicates per experiment. In total, there were 8,367,769,624 and 15,059,232,696 mapped reads, with an average of 236 million and 186 million reads per bio-replicate in human and mouse, respectively. (*) Reference for the RNA-seq data files (FASTQ and BAM).

Human				Mouse		
**Sample**	**Cell Line**	**Tissue**	**GEO Accession***	**Sample**	**Tissue**	**GEO Accession***
				Adipose : Genital FatPad : 8 Weeks	Adipose	GSM900190
				Adipose : Subcutaneous FatPad : 8 Weeks	Adipose	GSM900191
				Adrenal : 8 Weeks	Adrenal	GSM900188
				Bladder : 8 Weeks	Bladder	GSM1000564
Blood: Early Myeloid Progenitor	K562	Blood	GSM765405			
Blood: B lymphocyte	GM12878	Blood	GSM758559			
Blood: Monocyte	CD14+	Monocytes	GSM984609			
Blood: B Cells	CD20+	B Cells	GSM981256			
Brain: Epithelial: neuroblastoma	SK-N-SH	Brain	GSM981253	Brain : Cortex : 8 Weeks	Brain	GSM1000563
Brain: Epithelial: neuroblastoma	SK-N-SH-RA	Brain	GSM765395	Brain : Frontal Lobe : 8 Weeks	Brain	GSM1000562
				Brain : Cerebellum : 8 Weeks	Brain	GSM1000567
				Brain : Central Nervous System : E11.5	Brain	GSM1000573
				Brain : Central Nervous System : E14	Brain	GSM1000569
				Brain : Central Nervous System : E18	Brain	GSM1000570
				Brain : Whole Brain : E14.5	Brain	GSM1000572
Breast: Epithelial: adenocarcinoma	MCF7	Breast	GSM765388			
Cervix: Cervical Carcinoma	HeLa-S3	Cervix	GSM765402			
H1 Embryonic Stem Cells	H1-hESC	All	GSM758566			
				Heart : 8 Weeks	Heart	GSM900199
				Kidney : 8 Weeks	Kidney	GSM900194
				Large Intestine : Colon : 8 Weeks	Large Intestine	GSM900198
				Large Intestine : 8 Weeks	Large Intestine	GSM900189
				Limb : E14.5	Limb	GSM1000568
Liver: Epithalial: Hepatocellular Carcinoma	HepG2	Liver	GSM758575	Liver : E14	Liver	GSM1000574
				Liver : E14.5	Liver	GSM1000571
				Liver : E18	Liver	GSM1000566
				Liver : 8 Weeks	Liver	GSM900195
Lung: Fibroblast	IMR90	Lung	GSM981249	Lung : 8 Weeks	Lung	GSM900196
Lung: Fibroblast Cells	NHLF	Lung	GSM765394			
Lung: Epithelial: Carcinoma	A549	Lung	GSM758564			
Lung: Fibroblast	AG04450	Lung	GSM758561			
				Mammary Gland : 8 Weeks	MammaryGland	GSM900184
Muscle: Skeletal Muscle: Myoblasts	HSMM	Muscle	GSM758578			
				Ovary : 8 Weeks	Ovary	GSM900183
				Placenta : 8 Weeks	Placenta	GSM1000565
Skin: Fibroblast	BJ	Skin	GSM758562			
Skin: Epidermal Keratinocytes	NHEK	Skin	GSM765401			
				Small Intestine : Duodenum : 8 Weeks	Small Intestine	GSM900187
				Small Intestine : 8 Weeks	Small Intestine	GSM900186
				Spleen : 8 Weeks	Spleen	GSM900197
				Stomach : 8 Weeks	Stomach	GSM900185
				Testis : 8 Weeks	Testis	GSM900193
				Thymus : 8 Weeks	Thymus	GSM900192
Umbilical Cord	HUVEC	Umbilical Cord	GSM758563			

Supplementary Table 2: The list of transcript types corresponding to long transcripts.

Human	Mouse
3prime_overlapping_ncrna	IG_C_gene
IG_C_gene	IG_D_gene
IG_C_pseudogene	IG_J_gene
IG_D_gene	IG_V_gene
IG_J_gene	TEC
IG_J_pseudogene	ambiguous_orf
IG_V_gene	antisense
IG_V_pseudogene	disrupted_domain
TEC	lincRNA
TR_C_gene	ncrna_host
TR_D_gene	non_coding
TR_J_gene	nonsense_mediated_decay
TR_J_pseudogene	polymorphic_pseudogene
TR_V_gene	processed_pseudogene
TR_V_pseudogene	processed_transcript
ambiguous_orf	protein_coding
antisense	pseudogene
disrupted_domain	retained_intron
lincRNA	retrotransposed
ncrna_host	sense_intronic
non_coding	transcribed_processed_pseudogene
non_stop_decay	transcribed_unprocessed_pseudogene
nonsense_mediated_decay	unitary_pseudogene
polymorphic_pseudogene	unprocessed_pseudogene
processed_pseudogene	
processed_transcript	
protein_coding	
pseudogene	
retained_intron	
retrotransposed	
sense_intronic	
sense_overlapping	
transcribed_processed_pseudogene	
transcribed_unprocessed_pseudogene	
unitary_pseudogene	
unprocessed_pseudogene	

Supplementary Table 3: **Annotated and CAGE supported novel elements detected by RNA-seq in mouse (A), human (B) and both (C).** Elements are distinct exons, transcripts and genes. Annotated elements and novel transcripts are called detected if their IDR is smaller or equal to 0.1. Detected novel exons are distinct exons of detected novel transcripts, which do not overlap annotated exons on the same strand.

(A) Mouse

Gene category		Exons			Transcripts			Genes		
		Total	detected		Total	detected		Total	detected	
			Number	% of Total		Number	% of Total		Number	% of Total
Annotated	All long	345,616	327,381	94.7	90,100	75,967	84.3	31,915	27,184	85.2
	Protein-coding	320,024	309,131	96.6	78,261	69,364	88.6	22,380	20,494	91.6
	LncRNAs	16,107	12,964	80.5	5,669	3,742	66.0	3,845	3,207	83.4
	Other	9,599	5,390	56.2	6,170	2,861	46.4	5,690	3,483	61.2
Novel		Detected		Fold vs Annotated	Detected		Fold vs Annotated	NA		
		201,388		**0.58**	200,032		**2.22**			

(B) Human

Gene category		Exons			Transcripts			Genes		
		Total	Detected		Total	Detected		Total	Detected	
			Number	% of Total		Number	% of Total		Number	% of Total
Annotated	All long	509,579	406,630	79.8	164,174	106,572	64.9	43,575	29,279	67.2
	Protein-coding	432,261	375,287	86.8	131,409	97,121	73.9	20,007	18,341	91.7
	LncRNAs	49,513	20,839	42.1	17,547	5,386	30.7	10,840	5,451	50.3
	Other	29,635	12,183	41.1	15,218	4,065	26.7	12,728	5,487	43.1
Novel		Detected		Fold vs Annotated	Detected		Fold vs Annotated	NA		
		75,118		**0.15**	151,761		**0.92**			

(C) Both

Species	Annotated transcripts	Novel transcripts	Total transcripts
Mouse	90,100	200,032	**290,132**
Human	164,174	151,761	**315,935**

Supplementary Table 4: **Splice Junctions.** (A) Splice junctions by gene type. (B) Cross-classification of orthologous splice junctions.

(A) All detected SJ

	**Human**	**Mouse**
**Protein-coding**	277,953	289,405
**LncRNA**	13,281	11,133
**Pseudogene**	6,651	813
**Novel splice sites**	78,165	97,442
**Total**	**376,050**	**398,793**

(B) Orthologous SJ

		**Mouse**					
		**Protein coding**	**LncRNA**	**Pseudogene**	**Unassigned**	**Total**	**%**
**Human**	**Protein coding**	200,249	490	142	3	200,884	98.05%
	**LncRNA**	1,064	1,312	1	5	2,382	1.16%
	**Pseudogene**	1,212	9	18	0	1,239	0.60%
	**Unassigned**	68	251	6	57	382	0.19%
	Total	202,593	2,062	167	65	**204,887**	
	%	98.88%	1.01%	0.08%	0.03%		

Supplementary Table 5: **Conservation of antisense transcription.** (A) Contingency table of the number of genes with annotated antisense transcription in one-to-one orthologous gene pairs or with the antisense/total ratio greater than 30% in at least 70% of samples. Numbers in parentheses are the expected counts. Shown in boldface are the observed counts that are greater than the expected counts. (B) List of orthologous human and mouse protein-coding genes and their antisense, sharing a configuration similar to that described in[36].

(A)

		Mouse		
		AS	no AS	Total
Human	AS	**1,889** (1,098)	2,856 (3,647)	4,745
	no AS	1,752 (2,543)	**9,239** (8,448)	10,991
	Total	3,641	12,095	15,736

(B)

Human				Mouse			
Sense gene id	Sense gene name	Antisense gene id	Antisense gene name	Sense gene id	Sense gene name	Antisense gene id	Antisense gene name
ENSG00000175745	NR2F1	ENSG00000237187	RP11-65F13.2	ENSMUSG00000069171	Nr2f1	ENSMUSG00000087143	A830082K12Rik
ENSG00000108175	ZMIZ1	ENSG00000224596	RP11-202P11.1	ENSMUSG00000007817	Zmiz1	ENSMUSG00000087535	D930049A15Rik
ENSG00000197635	DPP4	ENSG00000230918	AC008063.2	ENSMUSG00000035000	Dpp4	ENSMUSG00000087518	Gm13561
ENSG00000168958	MFF	ENSG00000236432	AC097662.2	ENSMUSG00000026150	Mff	ENSMUSG00000085879	C430014B12Rik
ENSG00000052841	TTC17	ENSG00000254907	RP11-484D2.2	ENSMUSG00000027194	Ttc17	ENSMUSG00000045464	2810002D19Rik
ENSG00000178307	TMEM11	ENSG00000235530	AC087294.2	ENSMUSG00000043284	Tmem11	ENSMUSG00000091753	Gm17432
ENSG00000128585	MKLN1	ENSG00000231721	AC058791.2	ENSMUSG00000025609	Mkln1	ENSMUSG00000086212	Gm13845
ENSG00000154277	UCHL1	ENSG00000251173	RP11-124A7.2	ENSMUSG00000029223	Uchl1	ENSMUSG00000087601	Gm16832
ENSG00000111961	SASH1	ENSG00000224658	RP11-631F7.1	ENSMUSG00000015305	Sash1	ENSMUSG00000091633	Gm17280
ENSG00000135100	HNF1A	ENSG00000241388	HNF1A-AS1	ENSMUSG00000029556	Hnf1a	ENSMUSG00000086054	Gm13824
ENSG00000120896	SORBS3	ENSG00000251034	RP11-582J16.4	ENSMUSG00000022091	Sorbs3	ENSMUSG00000085557	Gm16600
ENSG00000107758	PPP3CB	ENSG00000221817	RP11-137L10.6	ENSMUSG00000021816	Ppp3cb	ENSMUSG00000084925	1810062O18Rik
ENSG00000123562	MORF4L2	ENSG00000231154	RP5-1055C14.7	ENSMUSG00000031422	Morf4l2	ENSMUSG00000087368	BC065397
ENSG00000163638	ADAMTS9	ENSG00000241684	ADAMTS9-AS2	ENSMUSG00000030022	Adamts9	ENSMUSG00000087573	9530026P05Rik
ENSG00000118197	DDX59	ENSG00000232257	RP11-92G12.3	ENSMUSG00000026404	Ddx59	ENSMUSG00000086553	9230116N13Rik
ENSG00000134107	BHLHE40	ENSG00000235831	AC018816.4	ENSMUSG00000030103	Bhlhe40	ENSMUSG00000087341	0610040F04Rik

Supplementary Table 6: **Classification of SINE elements in the 16 sense-antisense gene pairs with an inverted SINE in both human and mouse.**

Human PC gene	Human AS	Mouse PC gene	Mouse AS	Human SINE	Mouse SINE
ENSG00000232257	ENSG00000118197	ENSMUSG00000026404	ENSMUSG00000086553	AluSx<br>AluSq2	B3
ENSG00000221817	ENSG00000107758	ENSMUSG00000021816	ENSMUSG00000084925	AluSc<br>AluJo	MIRc
ENSG00000224658	ENSG00000111961	ENSMUSG00000015305	ENSMUSG00000091633	MIRb	MIR
ENSG00000230918	ENSG00000197635	ENSMUSG00000035000	ENSMUSG00000087518	MIR	PB1D9
ENSG00000235530	ENSG00000178307	ENSMUSG00000043284	ENSMUSG00000091753	AluSz6	B3
ENSG00000236432	ENSG00000168958	ENSMUSG00000026150	ENSMUSG00000085879	AluSx1<br>MIR	ID_B1
ENSG00000241388	ENSG00000135100	ENSMUSG00000029556	ENSMUSG00000086054	AluJb<br>AluSx<br>MIRb	B4A
ENSG00000251034	ENSG00000120896	ENSMUSG00000022091	ENSMUSG00000085557	AluSc5	B1_Mus2
ENSG00000251173	ENSG00000154277	ENSMUSG00000029223	ENSMUSG00000087601	MIRb	B3
ENSG00000254907	ENSG00000052841	ENSMUSG00000027194	ENSMUSG00000045464	AluJr	B1F1
ENSG00000224596	ENSG00000108175	ENSMUSG00000007817	ENSMUSG00000087535	MIRc<br>MIR3<br>MIR	RSINE1
ENSG00000231154	ENSG00000123562	ENSMUSG00000031422	ENSMUSG00000087368	MIRb	B3A
ENSG00000231721	ENSG00000128585	ENSMUSG00000025609	ENSMUSG00000086212	AluSx	ID4_
ENSG00000235831	ENSG00000134107	ENSMUSG00000030103	ENSMUSG00000087341	AluSg4<br>AluSx3	PB1D10
ENSG00000237187	ENSG00000175745	ENSMUSG00000069171	ENSMUSG00000087143	AluSc	B1_Mus2
ENSG00000241684	ENSG00000163638	ENSMUSG00000030022	ENSMUSG00000087573	AluJr	B1_Mm

Supplementary Table 7: **Summary table of histone modification data.** GEO accession numbers of ChIP-seq profiles used to compute the average histone modification levels in promoter regions in human (A) and in mouse (B). When multiple GEO accessions are listed, the bigwig tracks were pooled together for each combination of the antibody and cell line or tissue.

(A)

	H3K27ac	H3K36me3	H3K4me3
**A549**			GSM945244
**AG04450**	GSM1010912		GSM945177
**BJ**		GSM945207	GSM945178
**GM12878**	GSM733771	GSM733679,GSM945212	GSM733708,GSM945188
**H1HESC**	GSM733718	GSM733725	GSM733657
**HELAS3**	GSM733684	GSM733711,GSM945230	GSM733682,GSM945201
**HEPG2**	GSM733743	GSM733685,GSM945211	GSM733737,GSM945182
**HSMM**	GSM733755	GSM733702	GSM733637
**HUVEC**	GSM733691	GSM733757,GSM945233	GSM733673,GSM945181
**K562**	GSM733656	GSM733714,GSM945302	GSM733680,GSM945165
**MCF7**	GSM945854		GSM945269
**NHEK**	GSM733674	GSM733726,GSM945174	GSM733720,GSM945175
**NHLF**	GSM733646	GSM733699	GSM733723,GSM945262
**SKNSH_RA**		GSM945209	GSM945202

(B)

	H3K27ac	H3K36me3	H3K4me3
**CEREBELLUM_ADULT8WKS**	GSM1000097		GSM769027
**CORTEX_ADULT8WKS**	GSM1000100		GSM769026
**HEART_ADULT8WKS**	GSM1000093	GSM1000130	GSM769017
**HEART_E14.5**	GSM1000137		GSM1000135
**KIDNEY_ADULT8WKS**	GSM1000092	GSM1000063	GSM769016
**LIMB_E14.5**	GSM1000107		GSM1000086
**LIVER_ADULT8WKS**	GSM1000140	GSM1000151	GSM769014
**LIVER_E14.5**	GSM1000113		GSM1000110
**LUNG_ADULT8WKS**			GSM769012
**PLACENTA_ADULT8WKS**	GSM1000134		GSM1000132
**SMINTESTINE_ADULT8WKS**	GSM1000084	GSM1000069	GSM1000083
**SPLEEN_ADULT8WKS**	GSM1000138	GSM1000070	GSM769036
**TESTIS_ADULT8WKS**	GSM1000081	GSM1000067	GSM1000079
**THYMUS_ADULT8WKS**	GSM1000103	GSM1000068	GSM1000101
**WHOLEBRAIN_E14.5**	GSM1000094	GSM1000072	GSM1000095

Supplementary Table 8: **Published housekeeping gene sets and their intersection.** Only genes with a Gencode v10 id are considered.

HK gene set	identifier	Technique used	Number of genes in Gencode v10
Fantom5, Nature, 2014	F5	CDNA 5' end sequencing	6,560
Eisenberg et al., Trends in Genetics, 2013	E-L	RNA-seq	3,664
Chang et al., PLoS One, 2011	Chang	microarray	1,989
She et al., BMC Genomics, 2009	She	microarray	1,382
**Intersection**			**429**

Supplementary Methods

Genomes and annotation sets

Throughout this work we used Feb. 2009 assembly of the human genome (hg19, GRCh37) and Jul. 2007 assembly of the mouse genome (mm9, GRCm37) [1]. Human Gencode v10 and mouse ENSEMBL v65 databases were used for transcript annotations. Additionally, we considered a category of long transcripts composed of transcript types listed in Supplementary Table 2. Summary statistics on the annotated elements such as transcripts, genes and exons are listed in Supplementary Table 3A,B. In addition, genomes were partitioned into a disjoint union of exonic, intronic, and intergenic regions and, apart from it, into a disjoint union of genic and intergenic regions based on the annotated transcript sets [2]. Exonic regions were given priority over intronic regions; exonic, intronic, and genic regions were given priority over intergenic regions.

RNA-seq data processing

Mapping

RNA-seq reads were aligned to the human (hg19) and mouse (mm9) genomes using the STAR 1.9 software [3]. Up to 10 mismatches per paired alignment were allowed. Only alignments for reads mapping to 10 or fewer loci were reported. Annotations were not utilized for mapping the data. Mapped reads were used to generate contigs, splice junctions, de-novo transcript models and quantification of the annotation as described in [2] (see also below). Uniquely mapped reads were selected by *bamflag* software (http://github.com/pervouchine/bamflag). The Human Body Map (HBM) unstranded paired-end RNA-seq dataset (16 tissues, 50-nt reads) was also processed in the same way and used to complement human ENCODE in summary statistics section. Since HBM data was generated by a different protocol with shorter reads, not stranded, and without bio-replicates, we decided not to include it in any further analysis together with ENCODE cell line data.

Ascertainment of reproducibility

Non-parametric IDR (npIDR) ascertains reproducibility of the detection of genomic elements (such as splice junctions, exons, transcripts, etc) in RNA-seq experiments with biological replicates, referred to as 1 and 2 below. The elements in each bio-replicate are binned according to their signal, and for all bins the npIDR1in2 is calculated as the proportion of elements in each bin in replicate 1 that have exactly zero signal (i.e. not detected) in replicate 2. Similarly, the npIDR2in1 is calculated as the proportion of elements in each bin in replicate 2 that have exactly zero signal (i.e. not detected) in replicate 1. The final npIDR value for each bin is defined as the mean of npIDR1in2 and npIDR2in1. In the main manuscript and in this supplementary information, we will use IDR instead of npIDR.

Contig generation

Contigs represent regions of directional RNA-seq coverage. They are called from merged biological replicates but each contig is scored against individual replicates to facilitate IDR analysis. Contigs are required to have non-zero signal in both replicates. Only uniquely mapping reads are used for building and quantifying contigs. Neighboring contigs are merged if the gap between them is smaller than 25 bases. Contigs are strand-specific, but contigs with more than 9 times more antisense than sense signal are filtered as possible artifacts of strand-specific library construction. Each contig is associated with the following values: (1) BPKM, "Bases per Kilobase per Million mapped bases", averaged between the replicates; (2) a non-parametric irreproducible discovery score (npIDR); (3) the total number of mapped bases in the contig in both replicates (sum of wiggle track signal). Generation of contigs is independent of annotations.

TSS and CAGE support

Since *de novo* transcript models are less reliable than annotated transcripts, we only consider the ones whose TSS (most 5' bp) is supported by CAGE data from the most recent and diverse FANTOM study [4]. For this we used the 217,572 human and the 129,466 mouse TSS-like classified CAGE peaks identifies by FANTOM5 (http://fantom.gsc.riken.jp/5/tet).

More precisely de-novo transcript models' TSS is extended by 50 bp in both the 5' and the 3' directions, and the resulting 101bp segment is intersected with the TSS-like CAGE peaks. When an intersection in found, the *de novo* transcript model is considered supported by CAGE.

De novo transcript models

Cufflinks 1.0.3 [5] was used to assemble the transcripts from STAR alignments. Only uniquely mapping non-duplicated alignments crossing GU/AG junctions were utilized. The alignments from the two bio-replicates were merged before Cufflinks assembly. The Cufflinks gene, transcript and exon RPKM were quantified using Flux Capacitor [6] in each bio-replicate, and the resulting RPKM were assessed for reproducibility using npIDR [2]. Cufflinks models with an IDR value lesser or equal to 0.1 were further merged using *compmerge* (http://big.crg.cat/services/compmerge). The resulting merged cufflinks model intron chains were further compared to the annotated spliced transcript intron chains (using *comptr* available here: http://genome.crg.es/~sdjebali/Programs/comptr), and the spliced cufflinks models whose intron chain was neither equal nor included in the intron chain of an annotated transcript were kept. To obtain an even more complete and reliable set of novel transcripts, we further required the TSS of these transcripts to be supported by CAGE (see above). This resulted in sets of 200,032 novel transcripts in mouse and of 151,761 novel transcripts in human. Their exons were called novel if they did not overlap any annotated exon on the same strand (Supplementary Table 3).

The complete set of merged cufflinks models for each species is available in Supplementary data archive 1.

Average read density

Genome-wide read density was computed by *genomeCoverageBed* utility with -split and -bg options using only uniquely-mapped reads, separately for each bio-replicate and for each strand [7]. The outputs were combined into a single bedgraph file by *unionBedGraphs* utility separately for each strand [7]. Next, read density statistics were averaged genome-wide over consecutive 100-nt bins in each bio-replicate resulting in

20,580,077 and 20,623,806 such bins with at least one non-zero value in human and mouse, respectively (Supplementary Fig. 4A). The combined 100-nt averages from the plus and from the minus strand were normalized to have the same total read density and assessed for reproducibility between bio-replicates at IDR≤0.1 (Supplementary Fig. 4B). The average read density (i.e., average height of the pile of aligned reads, per 100 nt) was computed for each sample as the mean between bio-replicates or set to zero for bins, which did not pass the reproducibility filter. The average and the standard deviation of read density, taken across samples, were used as measures of center and spread of transcriptional activity, respectively; all probability distributions were computed for their base-10 logarithm transformations.

Common RNA

The amount of common RNA of a gene was defined to be the smallest of its RPKM values in a given set of samples. The amount of common RNA in a pair of samples was defined to be the sum of the amounts of common RNA of each gene as a fraction of the average (between samples) sum of gene RPKM values.

Quantification of histone modification levels

The bigWig whole-genome human and mouse ChIP-seq profiles were obtained from the Encode DCC portal (http://hgdownload.soe.ucsc.edu/goldenPath/, folders hg19/ and mm9/, respectively) by GEO accession numbers that are listed in Supplementary Table 7. We considered only data without additional chemical treatment.

Conservation of histone marks

The ChIP-seq profiles were processed by using bigWigAverageOverBed utility over ±500 nt windows centred at TSS (for H3K4me3 and H3K27ac) or at TTS (for H3K36me3) of annotated human and mouse protein-coding genes. Multiple bigWigs were pooled for each histone mark and condition. For each combination of ChIP-seq antibody and cell

line/tissue, the histone modification signals were normalized to have the same area under the curve (i.e., the signal in each TSS was divided by the sum of signals in all TSS for each given experiment). Next, the normalized densities were averaged across conditions for each TSS or TSS and ChIP-seq antibody and the corresponding gene expression values were also averaged over the same set of conditions. The results were matched between human and mouse according to the gene ortholog list. As before, zero values were replaced by the effective value of 10^{-3}.

Splicing quantification and analysis

The quantitative assessment of splicing at the level of splice junctions (SJ) was done by using intron-centric metrics as they allow to interrogate a broad range of splicing events, not only single-cassette exons [8]. The percent-spliced-in index ψ_5 (ψ_3) estimates the conditional probability of a SJ, i.e., the number of transcripts spliced from the donor site D to the acceptor site A relative to the number of transcripts in which D (respectively, A) was used as a splice site. The completeness of splicing index θ_5 (θ_3) estimates the respective absolute probability, i.e., the likelihood that splicing at D (respectively, A) has occurred.

Splice junction counts were quantified directly from short read alignments (see Mapping section) by using *sjcount* software (https://github.com/pervouchine/sjcount) as a part of the *ipsa* package (https://github.com/pervouchine/ipsa). The intron-centric metrics were computed by

1. considering only uniquely-mapped reads;
2. requiring the margin of 10 nt for every exon-exon as well as exon-intron junction;
3. requiring the minimum entropy of 3 bits for the offset distribution;
4. requiring agreement on SJ counts between bioreplicates (IDR<0.1);
5. requiring the minimum count of 10 in the denominator of the fraction defining ψ_5, ψ_3, θ_5 and θ_3 (see [8]).

Splice junctions were classified into the following four categories (increasing confidence) according to their annotation status:

1. Novel (both splice sites unannotated);
2. One of the two splice sites is annotated;
3. Both splice sites are annotated but the intron between them is not;
4. Both splice sites and the intron between them are annotated.

The abundance of splice junctions in each category, expressed as the number of splice junctions with count of at least *x* in at least *y* samples, is shown in Supplementary Fig. 1 (mouse). Each splice junction from categories 2-4 (see above) was assigned gene type (protein-coding, lncRNA, or pseudogene) according to the gene type of the annotated splice site. In cases when one splice site corresponded to several gene types or the two splice sites were assigned different gene types, protein-coding type was preferentially used over lncRNA, and lncRNA over pseudogene. The rest of splice junctions (category 1) correspond to unannotated splice sites. The categorization of human and mouse splice junctions is shown in Supplementary Table 4A.

Pooled values of ψ_i and θ_i (i=5,3), denoted by ψ and θ, respectively, were used to compute sample statistics. At that, ψ and θ samples with more than 25% missing values were excluded from the analysis. The average and the standard deviation of ψ and θ across samples were chosen to be the measures of center and spread of SJ's usage and processivity, respectively; all probability distributions were computed after applying the logistic transformation $logit(x)=log_{10}(x/(1-x))$.

An annotated splice site (respectively, SJ) is classified as *constitutive* if it appeared as a splice site (respectively, SJ) in all annotated transcripts overlapping the corresponding nucleotide range; otherwise it is classified as *alternative*. Approximately 40% of human and 20% of mouse SJ are alternative, likely due to the difference in the annotation depth. However, the fraction of human alternative SJ rises to 65% given that the mouse ortholog is alternative, revealing strong association between human and mouse annotations ($p<10^{-16}$). In contrast with the alternative usage, which by definition refers to the annotated SJ status,

in Figure 3B we refer to the variability of splicing metrics that was quantified from the RNA-seq data.

Non-coding genes

Long non-coding RNAs

Long non-coding RNAs (lncRNAs) are an emerging family of RNAs that have been shown to have a diverse spectrum of functionality [9,10]. As a general property, lncRNAs are highly lineage-specific and appear to have a rapid evolutionary turnover [11,12]. The GENCODE consortium has recently annotated a large collection of lncRNAs in the human genome [11]. There are 10,840 annotated human lncRNAs listed in Gencode v10. This compares to only 3,854 mouse lncRNAs annotated in ENSEMBL v65. Of these, we have detected 3,297 human and 3,207 mouse lncRNAs to be transcribed in the samples analyzed here. The larger detection rate for mouse lncRNAs is likely to be due to the broader biological spectrum of mouse samples. While the functionality of many lncRNAs is currently under discussion, it is generally accepted that lncRNAs conserved over large evolutionary distances are likely to play an important biological role.

Pseudogenes

Pseudogenes are a distinct class of long non-coding genes whose expression is encountered in selective cell types. Pseudogenes are distinguished from other non-coding genes because their parent protein-coding gene is found in the genome. A total of 12,358 human pseudogenes (from Gencode v10) and 15,887 mouse pseudogenes (identified *in silico* using Pseudopipe [13] based on ENSEMBL v65) have been identified. We have detected expression in 1,441 human and 878 mouse pseudogenes in the samples analyzed here — a proportion much lower than for lncRNAs. Of the several thousand pseudogenes, only 129 pseudogenes were found to be orthologs between human and mouse. The low percentage of orthologs in pseudogenes compared to that in protein-coding genes and lncRNAs is consistent with the retro-transposition burst events that happened after the speciation of human and mouse, which gave rise to a large number of pseudogenes independently in the two species [14]. We found that the parents of the orthologous pseudogenes are enriched for

housekeeping genes whose functions are related to cellular respiration and metabolism. Of the 129 orthologous pseudogenes, 27 mouse pseudogenes and 19 human pseudogenes are transcribed, where only 5 of the orthologs are transcribed in both species — a transcriptional behavior quite contrasting with that of lncRNAs.

Ortholog lists

Homology-based pipeline for lncRNAs

PipeR is a pipeline developed to profile lncRNAs applying an homology strategy that combines a set of similarity and aligning methods to detect the presence of the queries in a set of target genomes, as previously described in [11,15]. We further require the lncRNA transcript predicted in the target genome to cover at least 70% of the original query sequence, and to contain less than 20% of ancestral repeats in order to avoid the inclusion of non-related sequences that could lead to spurious mapping and predictions. The genomes are masked using *RepeatMasker* (http://www.repeatmasker.org) for low complexity and interspersed repeats.

Orthologs of protein-coding genes

Orthologs of protein-coding genes between human and mouse was obtained as described in [16] and [17]. This ortholog set includes 15,736 one-to-one orthologs, 1,527 one-to-many orthologs and 2,593 many-to-many orthologs. Throughout this paper, the analyses in matched gene pairs were made for one-to-one orthologs with long transcripts (Supplementary Table 2), representing a total of 15,722 genes.

Orthologs of novel transcripts

We used the novel mouse cufflinks models (see *de-novo* transcript model section) to discover novel 1-to-1 orthologous transcripts between mouse and human. Since novel transcripts strandedly overlapping the annotation are more complicated to deal with, novel mouse cufflinks models that were either intergenic or antisense (IA) to the annotation (using a 1bp overlap), were used. To discover orthology relationship they were first given as input to *pipeR* to find human orthologous transcripts, and those were further fed back to

pipeR to find 1-to-1 orthology relationship (for more details on the method see LncRNA ortholog supplementary section). Doing so we were able to identify 486 1-to-1 orthologs from the initial 4,094 mouse IA transcripts with CAGE support. Since we wanted to identify new transcripts in both species, we further discarded 155 transcripts with stranded exonic overlap with the human annotation, and 192 transcripts with stranded exonic overlap with the novel human cufflinks models. We also further required the transcripts to have CAGE support at their 5' end (see above) and ended up with a set of 38 novel 1-to-1 orthologous transcripts between mouse and human.

The 38 1-to-1 novel orthologous transcripts can be found in Supplementary data archive 1.

Orthologous genomic bins

The genomic sequences of human and mouse were subdivided into consecutive 100-nt bins. The midpoint of each 100-nt bin in the human genome was mapped to the mouse genome by a custom lift-over procedure [18] using filtered chain alignments [19]. The mapping of midpoints induced the mapping of 100-nt bins, to which they belong. The corresponding bins in the two species were declared as orthologous if they were mapped bijectively under this procedure, i.e., if the human-to-mouse and mouse-to-human induced mappings were mutually inverse as functions. Bins that were not identified as orthologous (i.e., ones that were either not mapped or not mapped uniquely) were discarded from further analysis. Although this procedure is biased towards considering genomic bins that are conserved, it captures many bins with low and intermediate phastCons conservation scores (median=2.0; IQR=9.1, min=0, max=100).

LncRNA orthologs

Human lncRNAs from Gencode v10 were mapped onto the mouse genome using the *PipeR* pipeline described above. More precisely we first mapped the 17,547 transcripts belonging to the 10,840 human Gencode lncRNA genes to the mouse genome, and obtained 2,327 transcripts (1,679 genes) in mouse, corresponding to 5,067 transcripts (3,887 genes) in human. This represents a set of one-to-many lncRNA ortholog transcripts between human and mouse, so we proceeded with further analysis of our predictions and used

pipeR to derive a subset of one-to-one orthologs by applying a reciprocal search to all of our predictions. As a result we were able to obtain 1,719 transcripts (1,277 genes) in mouse that were one-to-one orthologs. To have a comparison of ortholog conservation we also applied our strategy to find Gencode v10 lncRNAs in 4 other mammalian species (cow, dog, pig, rat). Similarity between the original human Gencode query and predictions in mouse are evaluated and scored as the percent identity of the alignment. The latest version of the pipeline can be found at http://github.com/cbcrg/piper-nf

The predicted LncRNAs from the mapping of Gencode V10 on mouse mm9 assembly were clustered into genes by stranded overlap of at least 1bp. The new set of transcript clusters was classified by taking the overlap with the set of en65 mouse long genes. If a transcript cluster overlapped on the same strand with an annotated gene then we assigned the annotated gene identifier to the transcript cluster. If there was no overlap we kept an identifier generated by the clustering. In the case of a transcript cluster overlapping with more than one annotated long gene the prefix "MO_" (multiple overlap) was added to the gene identifier. This merged annotation was further submitted to the Flux Capacitor [6] program for quantification, and the resulting RPKM values in each bioreplicate of a sample were submitted to npIDR.

To obtain a true 1-to-1 orthology at the gene level, we further filtered out all mouse genes corresponding to more than one human gene, and all sets of mouse genes coming from a single human gene. This lead to a set of 851 1-to-1 orthologous genes, including 1,083 transcripts in mouse, which can be found in Supplementary data archive 4.

To measure level of conservation of mouse lncRNA orthologs, the same homology strategy was used to find orthologs in other species. Human Gencode V10 were therefore mapped on 4 other mammalian species: rat (Rattus norvegicus, rn5), pig (Sus scrofa, Sscrofa10), dog (Canis familiars, CanFam3) and cow (Bos taurus, UMD3.1), using the exact same pipeline as for mouse. We then categorized the average gene expression in mouse (computed on the samples for which RPKM was higher than 0.1) according to the number of species in which an ortholog was found, and detected a weak correlation between both (Supplementary Fig. 3A).

Using npIDR 0.1 as a maximum threshold to call expression and requiring the gene to be expressed in at least 50% of the samples in mouse and 50% of the samples in human,

we found 12 genes, called ubiquitous. Compared to the set of 851 1-to-1 orthologous genes, these 12 genes had a much higher average ratio of nuclear vs cytosolic expression across 7 human cell lines (Supplementary Fig. 3A). This ratio was computed in each cell line by only considering the genes which had a non-zero RPKM after filtering by npIDR 0.1.

Pseudogene orthologs

To identify pseudogene orthologs, we started with 12,358 human pseudogenes from Gencode v10 and 15,887 mouse pseudogenes based on ENSEMBL v65, generated by *Pseudopipe* [13]. Each human pseudogene was mapped to the mouse genome using the filtered pairwise whole-genome human-mouse chain alignments [19], and similarly, each mouse pseudogene was mapped to the human genome. Orthologous human and mouse pseudogenes that overlapped by a minimum of 1bp in the defined syntenic regions were identified. From this set, we further filtered out those pseudogene pairs derived from non-orthologous parents, or of different biotypes (e.g., one as duplicated pseudogene and one as processed pseudogene), or of one-to-many mappings. This resulted in the identification of 129 one-to-one human-mouse pseudogene orthologs.

The resulting list of pseudogene orthologs can be found in supplementary data archive 4.

Orthologous SJs

Genomic positions of annotated splice sites in human and mouse were extracted from Gencode v10 and ENSEMBL65 annotations. Additionally, novel splice sites predicted from RNA-seq data were included in the analysis if (i) they were supported by non-zero SJ counts in at least 15% of samples and (ii) one of the boundaries of the SJ was annotated as splice site (i.e., the SJ with two unannotated boundaries were not considered).

Human splice sites were projected to the mouse genome by a per-nucleotide lift-over procedure [18] using filtered pairwise whole-genome chain alignments [19] and, similarly, mouse splice sites were projected to the human genome. Splice sites that were mapped uniquely and bijectively (i.e., the human-to-mouse and mouse-to-human projections were mutually inverse as functions) were said to be one-to-one orthologs. A human segment

(exons or introns) was said to be one-to-one orthologous to a mouse segment if the corresponding splice sites were orthologous (as defined above). In total, one-to-one correspondence was established for 203,039 and 202,259 pairs of donor and acceptor sites, respectively, and for 151,257 and 204,887 pairs of (internal) exons and introns, respectively (here the terms 'intron' and splice junction, SJ, are used interchangeably). One-to-one correspondence between splice sites induced an orthology relationship between human and mouse protein-coding genes, to which they belong. The induced relationship was identical to that of the human-mouse ortholog list in more than 93% of gene pairs [17], thus demonstrating validity of the approach. The splicing analysis pipeline is available at http://genome.crg.eu/~dmitri/splicing_pipelines/

The list of ortholog segments (exons and introns) is available as Supplementary data archive 2.

Constrained genes

DNR decomposition

The joint probability distribution of DNR and log-10 average gene expression was decomposed into the sum of two 2-dimensional Gaussian distributions corresponding to the two modes of the joint density (Supplementary Fig. 10A). Mean vectors and covariance matrices were estimated by the method of moments separately for each of the two modes [split by
the line log10(mean)=1.33(DNR-1)]. The weights of the two Gaussian components in the sum were estimated by computing the projection (in L2 norm) of the observed density onto a linear subspace generated by the two modes. The decomposition of DNR distribution (Supplementary Fig. 10B) was computed as a marginal distribution of 2D Gaussians.

Constrained gene tissue specificity

In each species, tissue specific genes are defined as genes in the top 20th percentile of the tissue specificity measure distribution, for all three following tissue specificity measures:

- Normalized entropy (nentropy)
- Coefficient of Variation (CV)
- Kendall tau index (tau).

Using this approach, we found 2,043 and 2,558 tissue specific genes in human and in mouse respectively, of which 990 and 726 are in the list of one-to-one orthologs with a DNR (see above), and of which 72 and 32 are constrained respectively. This means that 7.3% and 4.4% of human and mouse tissue specific genes that have a DNR, are constrained (respectively).

Constrained gene differential expression

In order to know whether constrained genes are more or less differentially expressed (DE) than the rest of the genes, we computed for each species and each pair of experiment, differentially expressed genes (EdgeR[20] on the read counts of the genes in the two pairs of bio-replicates, filtering by FDR ≤0.01 and by $\log_{10}$ fold-change ≥2). Since there are 18 and 30 experiments in human and in mouse respectively, this approach yielded 153 and 435 sets of differential expressed genes in human and mouse respectively. In total, 13,042 human and 14,567 mouse genes were found DE in at least one comparison,, representing 12,763 human and 13,550 mouse genes with DNR, and 5,467 human and 5,862 mouse constrained genes. This means that the percentage of genes with DNR that are DE is 89% for human and 99% for mouse, and that the percentage of constrained genes that are DE is 82% for human and 88% for mouse. This shows that constrained genes are DE at a rate that is similar to the one of orthologous genes with DNR.

Sets of constrained and unconstrained genes matched by expression

Constrained genes are globally more highly expressed than unconstrained genes, when considering all (Supplementary Fig. 11B), or only mouse or only human experiments (data not shown). Since this could potentially bias some analyses that compare constrained

and unconstrained genes, we also define equal size control sets of constrained and unconstrained genes with matched average expression (average expression being computed on non-zero RPKM after applying npIDR), using all, only mouse and only human experiments. This is done using an in-house tool which, given a set of elements associated to classes and values, samples from each class an equal number of elements so that the final distributions of values are similar between classes.

Applied to the sets of 6,636 constrained and 7,727 unconstrained genes, this procedure resulted in the following number of constrained and constrained genes of matched expression:

- 5,519, when the average is computed on all mouse and human experiments,
- 5,752, when the average is computed on human only experiments,
- 5,268, when the average is computed on human only experiments.

The set of 14,363 genes with DNR with information about constrained genes and gene sets matched by expression can be found in Supplementary data archive 5.

Constrained genes in vertebrates

In order to know whether genes with constrained expression in mouse and human were also constrained in other vertebrate species, we used gene expression data from two recently published multiple vertebrate and tissue RNA-seq studies:

- Merkin et al.[21], Science, 2013, including RNA-seq from 9 tissues (brain, colon, heart, kidney, liver, lung, skeletal muscle, spleen, testes) of 3 individuals from 5 vertebrate species (rhesus, mouse, rat, cow, chicken), complemented by the Human Body Map RNA-seq data from the same tissues. There were 6,002 orthologs across the 6 species, of which 5,971 had expression in both mouse and human using our data (Figure 3B);

- Barbosa et al.[22], Science, 2013, including RNA-seq from 7 tissues (brain, cerebellum, heart, kidney, liver, skeletal muscle, testes) and 11 vertebrate species (human, chimp, orangutan, macaque, mouse, opossum, platypus, chicken, frog, tetraodon).

Since there were an unequal number of tissues for each species, we chose 7 species (human, chimp, macaque, mouse, opossum, platypus, chicken) for which there were 6 tissues (brain, cerebellum, heart, kidney, liver, testes) available. The number of orthologs from those 7 species was 10,568.

Constrained splicing events

Following the same logic as for constrained genes, but instead of the dynamic range we compute the mean and the variance of SJ usage (ψ) for 204,887 orthologous SJs across the pooled set of human and mouse experiments. Since ψ value is not always defined and the value of variance is correctly defined only for large enough samples, we confine our analysis to 139,935 SJs that have at least two defined ψ values in each species. If the distribution of ψ were the extreme case, in which ψ be equal only to 0 or 1 (Bernoulli distribution), the variance of such distribution would have been equal to $\sigma^2=\mu^*(1-\mu)$, where μ is the proportion of ones among ψ. The actual distribution of ψ across experiments is continuous, in which the variable can also take intermediate values between 0 and 1. The variance of such observed distribution is smaller or equal to $\mu^*(1-\mu)$, where μ is the average value of ψ. We therefore represent the result on splicing constraint as absolute variance (Figure 6D) and the fraction of variance in the maximum possible variance of Bernoulli distribution with the same mean (Supplementary Fig. 14).

Nuclear versus cytosolic enrichment analysis

The analysis of nuclear vs. cytosolic enrichment was done using ENCODE cell line data [2] for protein-coding genes with non-zero expression values in nuclear, cytosolic, and cell compartments. The mean and standard deviation were computed on $\log_{10}$ of the nuclear-to-cytosolic concentration ratio for genes with observations in all seven ENCODE cell lines [2], separately for constrained and unconstrained genes matched by expression level (Figure 6A).

Gene ontology enrichment analysis

In order to know whether constrained genes were enriched in any particular GO term from any of the 3 main GO trees (biological process, molecular function, cellular compartment), we did a GO term enrichment analysis for the set of 6,626 constrained genes compared to the set of all 1-to-1 mouse/human orthologs using the GOstat R package for the three GO trees.

Results are provided in Supplementary data archive 5.

Broad promoter usage

In order to get insight into the gene regulation of constrained and unconstrained genes, we computed the number and percent of constrained and unconstrained genes with broad TSS as defined by FANTOM[4,23]. In order to eliminate the gene expression bias, this computation was done for the 5,268 constrained and the 5,268 unconstrained genes with matched expression in human. The percent was 67% for constrained genes and 52% for unconstrained genes (p-val≈0).

Transcription Factor peaks

The clustered peaks for ENCODE transcription factor binding were downloaded from the UCSC ftp site (http://hgdownload.cse.ucsc.edu/goldenpath/hg19/encodeDCC/wgEncodeRegTfbsClustered/wgEncodeRegTfbsClusteredV3.bed.gz), as genomic regions. We only count peaks overlapping by at least 1bp the TSS extended 2000 and 500 bp upstream and downstream, respectively.

Repeat elements

Since transcription initiating in retrotransposons have been shown to be cell type specific[24], we were interested in knowing whether promoters of unconstrained genes were enriched for repeat elements with respect to constrained genes. Therefore we computed the 1kb density of repeat elements (UCSC, 5,232, 244 repeats downloaded on Feb. 2013) at

the promoter of genes as well as in the gene body. The promoter of a gene was defined as the 1001 bp window centered at the TSS of the gene, whereas the body of a gene was defined as the segment between the two gene extremities extended by 500bp on each side.

We found that the mean-per-1kb repeat density at the promoter was 0.82 for the constrained and 0.87 for the unconstrained genes with the standard deviations 1.15 and 1.19, respectively. The p-value for two-sample z-test (n=5519, normality not required) was 0.03, indicating a depletion of repeat elements in promoter regions of constrained genes.

Comparison to HK genes

Housekeeping (HK) genes are usually defined as genes with little expression variation across many different cell types of an organism. Therefore our set of constrained genes could be seen as a mouse-human HK gene collection. For this reason we were interested in comparing it to several recently published sets of HK genes:

- 3,804 HK genes defined as genes with little expression variation across 16 human tissues using Human Body Map RNA-seq [25], called E-L HK genes here;
- 7,522 genes that we derived from the 10,787 HK TSS defined using single-molecule cDNA sequencing (CAGE) across a great diversity of human primary cells, cell lines and tissue [4], called F5 HK genes here;
- 2,064 HK genes obtained using microarray in 43 human tissues [26], called Chang HK genes here;
- 1,522 HK genes obtained using microarray in 42 normal human tissues [27], called She HK genes here.

To be compared to our set of constrained genes, which were defined in Gencode v10, the genes in each of those sets, first need to be mapped to Gencode v10 gene id list, resulting in 3,664 E-L HK genes, 6,560 F5 HK genes, 1,989 Chang HK genes and 1,382 She HK genes (Supplementary Table 8).

There were 2,487 genes in common between E-L, F5 and the constrained genes, and only 335 between the 5 sets. Focusing on the 3 deep-sequencing derived sets, the set with

more unique genes was the constrained set, then F5 and finally E-L (Supplementary Fig. 15).

Relation to lethality

To help understand the properties of mouse/human constrained genes with respect to rest of the mouse/human orthologous genes, we compared them to a public database of mouse genes which mutation in homozygous mouse embryos have been proven to cause lethality *in vivo* (Mouse embryonic lethal data from the Jax mice database[28], http://jaxmice.jax.org/list/ra50.html, called mouse lethal genes here). The July 3rd 2014 version of this database contained a total of 253 genes, of which 237 had a mouse ensembl v65 gene identifier.

A hundred and fifteen constrained genes were mouse lethal out of 6,636 (1.73%), while 101 unconstrained genes were mouse lethal out of 7,727 (1.31%). When focusing on the 1000 top constrained and the 1000 top unconstrained genes (i.e. with 1000 highest and lowest DNR), these numbers went to 24 (2.4%) and 9 (0.9%), therefore showing an even larger difference (Figure 6D).

Relation to traits and disease

In order to understand whether constrained and unconstrained behave differently with respect to certain traits/diseases, we compared them to data from the three following public databases:

- The Online Mendelian Inheritance in Man (OMIM) disease database (http://www.omim.org/downloads)[29], downloaded on June 20th 2014 and including 2,211 diseases, 3,090 genes and 4,820 associations;
- The NHGRI Genome-Wide Association Study (GWAS) catalog (www.genome.gov/gwastudies)[30], downloaded on June 20th 2014 and including 947 traits associated to known genes, 6,608 genes and 13,020 associations;
- The Catalog of Somatic Mutations in Cancer (COSMIC) (http://cancer.sanger.ac.uk/cancergenome/projects/cosmic/download)[31], downloaded on June 24th 2014 and including 48 cancer primary sites, 177 primary

histologies, 20,917 genes, 311 (primary site, primary histology) pairs, 318,132 associations between a (primary site, primary histology) pair and a gene.

For each database, 2 different analyses were performed:

1. the percent of genes in all and in the top 1000 constrained and unconstrained genes that were associated to a trait or disease were computed;
2. the number of traits or diseases that were significantly associated to the set of 6,636 constrained genes compared to the set of all 14,363 orthologous genes with DNR, using a hyper-geometric test and a p-value threshold of 0.01, was computed.

The results are summarized in Figure 6D. They show that unconstrained genes are more associated to postnatal diseases than constrained genes, consistent with the above lethality analysis, and this trend was even more important when using the top 1000 constrained and unconstrained gen. Although the number of significant OMIM diseases and GWAS traits were higher for unconstrained genes than for constrained genes, the number of significant cancer types was higher for constrained genes.

Relation to eQTL

In order to know whether mutations associated to expression change (eQTL) affect constrained genes more often than unconstrained genes, we downloaded and used two recently published sets of eQTLs:

- The 10,914 cis-eQTLs from the Battle et al. study [32];
- The 4,010,238 cis-eQTLs from the Lappalainen et al. study [33] (European population, FDR 5%).

Then we simply computed the percent of genes in all and in the top 1000 constrained and unconstrained genes that had an eQTL in each set. The results are summarized in Figure 6D. They show that the constrained genes have more eQTL than unconstrained genes.

Variation across human individuals

Constrained genes are defined as genes with low variation of expression across a diverse panel of mouse and human tissues and cell lines. It is therefore interesting to see whether those genes also vary less than other genes across human individuals.

To answer this question we used the Geuvadis gene expression data in 667 individuals (RPKM, see [33]) and computed the coefficient of variation of RPKM across the 667 individuals for both constrained and unconstrained genes (human matched expression set, see above). The results are shown on Supplementary Fig. 16 and indicate that constrained gene expression also varies less than unconstrained genes across human individuals.

Sequence conservation

PhastCons scores for multiple alignments of 45 vertebrate genomes to the human genome [34] were obtained from UCSC Genome browser database [1]. PhastCons scores for individual nucleotides were averaged over 100-nt windows and scaled to the range from 0 to 100.

The percent identity of promoter sequences was computed for the one-to-one protein-coding gene orthologs. Sequences 200bp upstream of the annotated transcription start site of the transcripts that define protein similarity were aligned using *T-coffee* program with default options [35]. The percent identity of promoter sequences was defined as the ratio of matching nucleotides to nucleotides that were aligned. Similarly, the percent identity of transcript sequences was computed by aligning transcripts that were used to define protein similarity, also as the ratio of matching nucleotides to nucleotides that were aligned. In most analyses, genes were categorized by their relative positions in the distribution of percent identity (i.e., top 20% conserved, etc.) by using the respective quantiles.

Antisense transcription

Antisense vs. total expression ratio

The antisense/total expression ratio was calculated for all genes in the long gene type classification (Supplementary Table 2). Specifically, for each gene we counted the number of reads mapping to the cognate strand (S), extended 1,000 nt upstream and

downstream, and the number of reads mapping to the opposite strand (AS). We retained only those counts that passed reproducibility filter (IDR<0.01). Then the ratio was computed as AS/(AS+S), for the genes with a total read count on both strands (AS+S) > 250.

The antisense/total expression ratio was averaged across all samples for each species, but only the genes with a valid ratio in at least 70% of the samples were considered. The correlation of the ratio between human and mouse was computed by taking the logit of the average ratio across samples. As a measure of divergence of the antisense/total expression ratio, we calculated the absolute difference between *logit* of the average ratio across samples in human and mouse.

Identification of orthologous sense-antisense pairs

Starting from the list of 1 to 1 orthologous protein-coding genes, we extracted the ones with an overlapping annotated long gene on the opposite strand, requiring at least 1 exonic nucleotide in common. For human and mouse, we found 4,286 and 3,181 protein-coding genes with this criteria, respectively. These genes were pooled with the orthologous protein-coding genes with an antisense vs total ratio > 30% in at least 70% of the conditions. This led to a final set of 4,745 human genes and 3,641 mouse genes, 1,889 of which have an orthologous relationship (Supplementary Table 5A).

Human and mouse genes with antisense transcription can be found in Supplementary data archive 3.

Specific examples of orthologous sense-antisense pairs

A regulatory mechanism involving antisense lncRNAs that contains a SINE elements and overlaps a protein-coding gene has been recently described [36]. Here we found 15 additional cases in which orthologous human/mouse protein-coding genes overlap SINE-containing lncRNA antisense transcripts (Supplementary Table 5B). Some of these cases exhibit coordinated lncRNA-mRNA expression both in human and in mouse (Supplementary Fig. 2A,B). Among these, we found HNF1A, a transcriptional activator that regulates the tissue-specific expression of multiple genes, specifically in pancreatic islet and in liver cells. In human, the expression of HNF1A and its lncRNA counterpart, HNF1A-AS1, is restricted to HepG2 and A549 (Supplementary Fig. 2A,C). In mouse, we also found

some potentially interesting cases such as the gene pair involving Bhlhe40, a gene encoding a transcription factor for neuronal differentiation for which the antisense gene shows neuronal tissue specificity (Supplementary Fig. 2B,D).

Intergenic transcription

Highly expressed intergenic bins

Intergenic bins are defined as mouse-human orthologous 100 bp bins with positive expression in one of the two species (see section on orthologous lists above) that do not overlap any Gencode v10 gene in human. Intergenic bins are considered as highly expressed when their expression (measured as average mean expression in human and mouse) is in the top 10 percentile of the intergenic bin expression distribution. Out of 6,610,763 orthologous 100bp bins with positive expression in human or mouse, 1,529, 377 are intergenic. Highly expressed intergenic bins were compared to GWAS hits[30] and to cis-eQTL[32] in order to see if they were enriched for those with respect to intergenic bins in general. Out of 152,937 highly expressed intergenic bins, 91 and 234 had at least one GWAS hit and one cis e-QTL respectively, compared to 770 and and 715 for intergenic bins in general, yielding p-values of 0.05 and nearly 0 respectively by Fisher test.

Supplementary Data

Supplementary data files and the supporting information can be found at the web site http://public-docs.crg.es/rguigo/Papers/Pervouchine_Nature_2014/SupplDataFiles/

Supplementary References

1 Meyer, L. R. *et al.* The UCSC Genome Browser database: extensions and updates 2013. *Nucleic Acids Res* **41**, D64-69, doi:gks1048 [pii]

10.1093/nar/gks1048 (2013).

2 Djebali, S. *et al.* Landscape of transcription in human cells. *Nature* **489**, 101-108, doi:nature11233 [pii]

10.1038/nature11233 (2012).

3 Dobin, A. *et al.* STAR: ultrafast universal RNA-seq aligner. *Bioinformatics* **29**, 15-21, doi:bts635 [pii]

10.1093/bioinformatics/bts635 (2013).

4 Forrest, A. R. *et al.* A promoter-level mammalian expression atlas. *Nature* **507**, 462-470, doi:nature13182 [pii]

10.1038/nature13182 (2014).

5 Trapnell, C. *et al.* Transcript assembly and quantification by RNA-Seq reveals unannotated transcripts and isoform switching during cell differentiation. *Nat Biotechnol* **28**, 511-515, doi:nbt.1621 [pii]

10.1038/nbt.1621 (2010).

6 Montgomery, S. B. *et al.* Transcriptome genetics using second generation sequencing in a Caucasian population. *Nature* **464**, 773-777, doi:nature08903 [pii]

10.1038/nature08903 (2010).

7 Quinlan AR, H. I. BEDTools: a flexible suite of utilities for comparing genomic features. *Bioinformatics* **26**, 841-842 (2010).

8 Pervouchine, D. D., Knowles, D. G. & Guigo, R. Intron-centric estimation of alternative splicing from RNA-seq data. *Bioinformatics* **29**, 273-274, doi:bts678 [pii]

10.1093/bioinformatics/bts678 (2013).

9 Mattick, J. S. The genetic signatures of noncoding RNAs. *PLoS Genet* **5**, e1000459, doi:10.1371/journal.pgen.1000459 (2009).

10 Ponting, C. P., Oliver, P. L. & Reik, W. Evolution and functions of long noncoding RNAs. *Cell* **136**, 629-641, doi:S0092-8674(09)00142-1 [pii]

10.1016/j.cell.2009.02.006 (2009).

11 Derrien, T. *et al.* The GENCODE v7 catalog of human long noncoding RNAs: analysis of their gene structure, evolution, and expression. *Genome Res* **22**, 1775-1789, doi:22/9/1775 [pii]

10.1101/gr.132159.111 (2012).

12 Young, R. S. & Ponting, C. P. Identification and function of long non-coding RNAs. *Essays Biochem* **54**, 113-126, doi:bse0540113 [pii]

10.1042/bse0540113 (2013).

13 Zhang, Z. *et al.* PseudoPipe: an automated pseudogene identification pipeline. *Bioinformatics* **22**, 1437-1439, doi:btl116 [pii]

10.1093/bioinformatics/btl116 (2006).

14 Ohshima, K. *et al.* Whole-genome screening indicates a possible burst of formation of processed pseudogenes and Alu repeats by particular L1 subfamilies in ancestral primates. *Genome Biol* **4**, R74, doi:10.1186/gb-2003-4-11-r74

gb-2003-4-11-r74 [pii] (2003).

15 Esteve-Codina, A. *et al.* Exploring the gonad transcriptome of two extreme male pigs with RNA-seq. *BMC Genomics* **12**, 552, doi:1471-2164-12-552 [pii]

10.1186/1471-2164-12-552 (2011).

16 The-mouse-ENCODE-consortium. A comparative encyclopedia of DNA elements in the mouse genome. *Nature*, doi:10.1038/nature13992 (2014).

17 Wu, Y.-C., Bansal, M. S., Rasmussen, M. D., Herrero, J. & Kellis, M. Phylogenetic identification and functional validation of orthologous genes

across human, mouse, fly, worm, yeast. *bioRxiv*, doi: http://dx.doi.org/10.1101/005736 (2014).

18 Pervouchine, D. D. *et al.* Evidence for widespread association of mammalian splicing and conserved long-range RNA structures. *RNA* **18**, 1-15, doi:rna.029249.111 [pii]

10.1261/rna.029249.111 (2012).

19 Denas, O. *et al.* Genome-wide comparative analysis reveals human-mouse regulatory landscape and evolution *bioRxiv*, doi:http://dx.doi.org/10.1101/010926 (2014).

20 Anders, S. *et al.* Count-based differential expression analysis of RNA sequencing data using R and Bioconductor. *Nat Protoc* **8**, 1765-1786, doi:nprot.2013.099 [pii]

10.1038/nprot.2013.099 (2013).

21 Merkin, J., Russell, C., Chen, P. & Burge, C. B. Evolutionary dynamics of gene and isoform regulation in Mammalian tissues. *Science* **338**, 1593-1599, doi:338/6114/1593 [pii]

10.1126/science.1228186 (2012).

22 Barbosa-Morais, N. L. *et al.* The evolutionary landscape of alternative splicing in vertebrate species. *Science* **338**, 1587-1593, doi:338/6114/1587 [pii]

10.1126/science.1230612 (2012).

23 Haberle, V. *et al.* Two independent transcription initiation codes overlap on vertebrate core promoters. *Nature* **507**, 381-385, doi:nature12974 [pii]

10.1038/nature12974 (2014).

24 Faulkner, G. J. *et al.* The regulated retrotransposon transcriptome of mammalian cells. *Nat Genet* **41**, 563-571, doi:ng.368 [pii]

10.1038/ng.368 (2009).

25 Eisenberg, E. & Levanon, E. Y. Human housekeeping genes, revisited. *Trends Genet* **29**, 569-574, doi:S0168-9525(13)00089-9 [pii]

10.1016/j.tig.2013.05.010 (2013).

26 Chang, C. W. *et al.* Identification of human housekeeping genes and tissue-selective genes by microarray meta-analysis. *PLoS One* **6**, e22859, doi:10.1371/journal.pone.0022859

PONE-D-11-07227 [pii] (2011).

27 She, X. *et al.* Definition, conservation and epigenetics of housekeeping and tissue-enriched genes. *BMC Genomics* **10**, 269, doi:1471-2164-10-269 [pii]

10.1186/1471-2164-10-269 (2009).

28 Laboratory, T. J. *Developmental Biology Research: Embryonic Lethality (Homozygous) - Jax mice strains*, <jaxmice.jax.org/list/ra50.html> (2014).

29 (McKusick-Nathans Institute of Genetic Medicine, Johns Hopkins University (Baltimore, MD), 2014).

[30] Welter, D. *et al.* The NHGRI GWAS Catalog, a curated resource of SNP-trait associations. . *Nucleic Acids Research* **42** (2014).

[31] Forbes, S. A. *et al.* COSMIC: mining complete cancer genomes in the Catalogue of Somatic Mutations in Cancer. *Nucleic Acids Res* **39**, D945-950, doi:gkq929 [pii]

10.1093/nar/gkq929 (2011).

[32] Battle, A. *et al.* Characterizing the genetic basis of transcriptome diversity through RNA-sequencing of 922 individuals. *Genome Res* **24**, 14-24, doi:gr.155192.113 [pii]

10.1101/gr.155192.113 (2014).

[33] Lappalainen, T. *et al.* Transcriptome and genome sequencing uncovers functional variation in humans. *Nature* **501**, 506-511, doi:nature12531 [pii]

10.1038/nature12531 (2013).

[34] Siepel, A. *et al.* Evolutionarily conserved elements in vertebrate, insect, worm, and yeast genomes. *Genome Res* **15**, 1034-1050, doi:gr.3715005 [pii]

10.1101/gr.3715005 (2005).

[35] Notredame, C., Higgins, D. G. & Heringa, J. T-Coffee: A novel method for fast and accurate multiple sequence alignment. *J Mol Biol* **302**, 205-217, doi:10.1006/jmbi.2000.4042

S0022-2836(00)94042-7 [pii] (2000).

[36] Carrieri, C. *et al.* Long non-coding antisense RNA controls Uchl1 translation through an embedded SINEB2 repeat. *Nature* **491**, 454-457, doi:nature11508 [pii]

10.1038/nature11508 (2012).
